# Progressive sub-arc mantle oxidation modulated by sediment melt

**DOI:** 10.1126/sciadv.aeb9023

**Published:** 2026-04-03

**Authors:** Mingdi Gao, Yu Wang, Yi-Gang Xu

**Affiliations:** ^1^ State Key Laboratory of Deep Earth Processes and Resources, Guangzhou Institute of Geochemistry, Chinese Academy of Sciences, Guangzhou 510640, China.; ^2^ Guangdong Research Center for Strategic Metals and Green Utilization, Guangzhou 510640, China.

## Abstract

The redox state of the sub-arc mantle beneath volcanic arcs is typically oxidized but exhibits substantial variability. Previous studies proposed that the incorporation of slab components alters both arc magma compositions and sub-arc mantle redox state, whereas the dominant component remains debated. Here, by compiling global Cenozoic primitive arc basalt and olivine-hosted melt inclusion data, we show that sediment melt dominated by terrigenous origin plays the controlling role. Progressive influx of sediment melts into the sub-arc mantle results in gradual enrichment of potassium oxide and most incompatible elements in arc magmas. Increases in potassium oxide content in primitive magmas also correlates with rising oxygen fugacity (*f*o_2_) values, highlighting the strong oxidizing potential of sediment melt. The high–potassium oxide, high-*f*o_2_ arc magmas spatially correlate with the distribution of Cenozoic porphyry copper-gold deposits, showing that the sediment melt influx not only elevates the sub-arc mantle *f*o_2_ but also enhances the metallogenetic potential for porphyry deposits.

## Teaser

Global variations in arc basalt composition and sub-arc mantle oxygen fugacity are dominated by the flux of sediment melt.

## INTRODUCTION

Arc magmas in subduction zones are generally more oxidized than those from nonsubduction settings, such as mid-ocean ridge basalts (MORBs) and ocean island basalts (*1*–*4*). This oxidized nature is a crucial precondition for the formation of porphyry deposits in subduction zones (*5*–*7*). While magma differentiation plays an important role in the oxidation of arc magmas (*7*, *8*), studies on primitive arc basalts and arc peridotites reveal that this oxidized characteristic can be inherited from an oxidized mantle source, largely due to the incorporation of oxidized slab components into the mantle (*2*, *9*, *10*). These oxidizing agents can be fluids and melts derived from the slab (*11*–*13*) or solid diapirs ascended into the mantle wedge (mélange) (*14*–*16*). Each agent exhibits varying oxidation capacities, resulting in substantial spatial heterogeneity in sub-arc mantle redox states, with oxygen fugacity (*f*o_2_) variations reaching up to one to two log units (*1*, *3*, *17*).

One hypothesis posits that oxidized sulfate carried by altered oceanic crust (AOC)– and serpentinite-derived fluids dominates the sub-arc mantle redox state variation, in which fluids released by cold slabs transport elevated oxidized sulfate fluxes into the sub-arc mantle, resulting in higher arc lava *f*o_2_ values in cold subduction zones (*18*–*20*). However, studies on natural samples argue that AOC- and serpentinite-derived fluids only contribute negligible sulfate to the oxidation of the sub-arc mantle (*21*–*23*). The sediment layer, on the other hand, may play a critical role in modulating the sub-arc mantle redox state from multiple perspectives. It can act as a redox filter, oxidizing AOC- and serpentinite-derived fluids (*24*), or may release fluids and/or melts with high oxidation capacities (*22*, *25*, *26*). Moreover, a data compilation of global arc basalts reveals that the sub-arc mantle *f*o_2_ is latitude dependent (*27*). They proposed that sediments deposited on low-latitude ocean floors contain more reduced organic carbon, resulting in less oxidized sub-arc mantle beneath low-latitude arcs. However, the deposition and preservation of organic carbon in sediments are rather complexing (*28*), and latitude is far from sufficient to account for the variations. For instance, a recent study has revealed no detectable dependence on latitudinally controlled biologic processes in arc magma systems (*29*).

These studies show that the primary causes of variations in sub-arc mantle redox states remain a topic of considerable debate. In this contribution, we compiled global Cenozoic primitive arc basalt data {MgO = 6 to 12 wt %, Mg# [molar 100 × Mg/(Mg + Fe)] = 60 to 72} and olivine-hosted melt inclusion data from arc lavas. On the basis of the compiled dataset, we calculated the *f*o_2_ values using whole-rock V/Sc ratios and host olivine-melt inclusion V partitioning. By assessing the magma composition and *f*o_2_ variations, we found that the influx of sediment melt dominated by terrigenous origin results in the enrichment of K_2_O and most incompatible element in magma, as well as the rising of *f*o_2_ recorded in these magmas. Continental arcs typically exhibit enhanced sediment melt contribution in their sub-arc mantle, resulting in elevated mantle *f*o_2_ values and increased metallogenetic potential for porphyry deposits within these arcs.

## RESULTS AND DISCUSSION

### Arc basalt compositional variations controlled by sediment melt

Arc basalts are generated by the partial melting of sub-arc mantle that has been metasomatized by various subducted slab components (*11*). These slab-derived components have distinct geochemical characteristics, particularly in their abundances of multivalent elements (e.g., S, Fe, and C), which, in turn, influence the composition and redox state (*f*o_2_) of arc magmas. To elucidate the *f*o_2_ variation, it is essential to identify the dominant factor(s) controlling the variability in arc magma compositions.

**Fig. 1.**
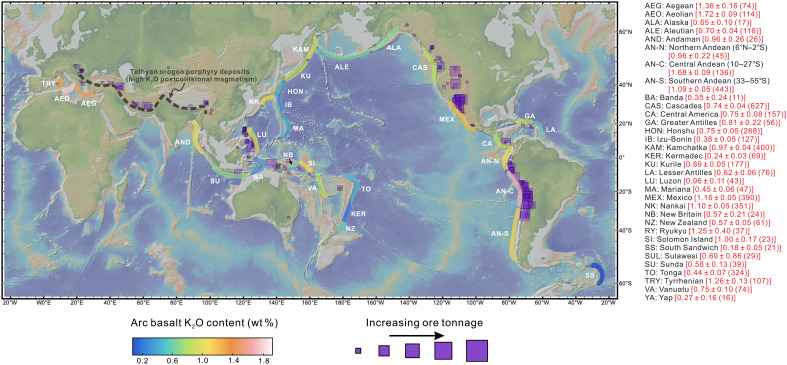
Spatial distribution of primitive arc basalts and Cenozoic porphyry Cu-Au deposits. Map symbols are colored on the basis of K_2_O content (in weight %), where high-K_2_O arc basalts mainly distribute in the eastern Pacific continental arcs. The red number in the right side is the averaged basalt K_2_O content in each arc segment and the 2 SEMs, with number of records in the bracket. The distribution and tonnage of global porphyry deposits are from Singer *et al.* (*111*). Abbreviations for arcs in are the same hereinafter.

**Fig. 2.**
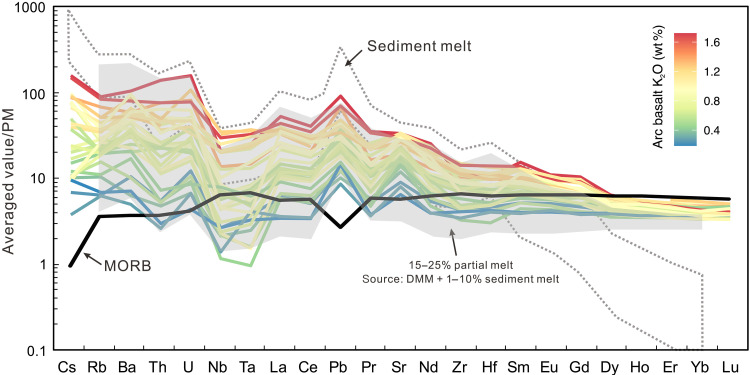
Trace element distribution patterns of global primitive arc basalts. Each line represents the averaged value in each arc segment. The line color indicates the averaged magma K_2_O content. Apart from HREE, most incompatible element content increases with increasing magma K_2_O content. Compositional range of the sediment melts is from the experimental study of Hermann and Rubatto (*48*). The MORB line is from the averaged primitive MORB compositions of Gale *et al.* (*100*) (data S2). The gray area represents the compositional range of partial melts from a DMM source hybridized by 1 to 10% sediment melt (see data S6 for calculation details). PM, primitive mantle.

Compared to MORBs, global primitive arc basalts show more scattered but generally consistent compositions for most major elements (fig. S1 and data S1 and S2). A key distinction is that arc basalts exhibit notably higher and more variable K_2_O contents and K_2_O/Na_2_O ratios (fig. S1), with K_2_O increasing from intraoceanic arcs (as low as 0.2 wt %) to continental arcs (up to 1.7 wt %) (Fig. 1), while MORBs typically have <0.2 wt % K_2_O. In terms of trace element compositions, the increase in K_2_O content is accompanied by a gradual enrichment of large-ion lithophile elements (LILEs), Th, U, Pb, light to middle rare-earth elements (LREEs to MREEs), and high-strength field elements (HFSEs) (Fig. 2 and data S1) (*30*). As these elements are highly incompatible during mantle melting, their pronounced enrichment in high-K_2_O continental arc magmas could arise from several processes, including crustal assimilation, relatively enriched mantle sources and/or low degrees of mantle melting, and the involvement of slab-derived components.

**Fig. 3.**
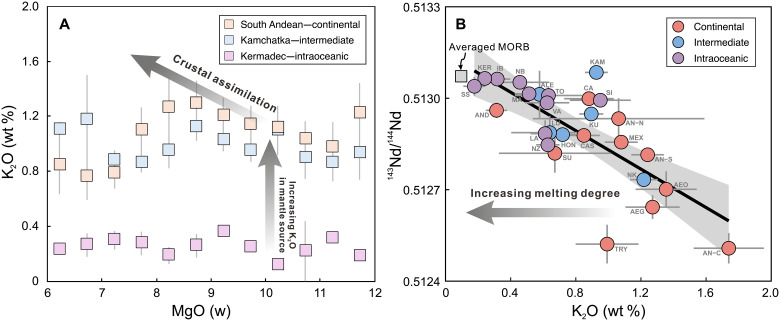
Influence of crustal assimilation and mantle melting degree on global primitive arc basalt K_2_O variations. The constant basalt K_2_O levels with decreasing MgO content in three representative arcs suggest that the effect of crustal assimilation is minimal (**A**). A negative correlation between K_2_O contents and ^143^Nd/^144^Nd values indicates that variations in arc basalt K_2_O content are not primarily determined by the degree of mantle melting (**B**). Error bars represent 2 SEMs. Classification of arc type (continental, intermediate, or intraoceanic) is from Frisch (*112*). Averaged MORB compositions here and hereinafter are the averaged primitive MORB compositions compiled from Gale *et al.* (*100*) (data S2).

First, during their ascent, continental arc magmas are more susceptible to assimilating K_2_O-rich continental crust materials, which could elevate K_2_O and other incompatible elements. However, in representative continental and intermediate arcs (e.g., Southern Andean and Kamchatka), arc basalts maintain consistently high-K_2_O contents as MgO decreases. This trend, when compared to intraoceanic arcs (e.g., Kermadec), indicates that the elevated K_2_O contents cannot be attributed solely to crustal assimilation (Fig. 3A).

Second, continental arc magmas are commonly considered to experience lower extents of mantle melting and/or to originate from a more enriched ambient mantle compared to intraoceanic arc magmas (*31*–*35*), which may lead to higher K_2_O and other incompatible element contents. Global arc basalt data display negative correlations between K_2_O contents and ^143^Nd/^144^Nd values (Fig. 3B). Similar negative correlations are observed for LILE (e.g., Ba), LREE to MREE (e.g., La and Sm), and HFSE (e.g., Nb and Zr) with ^143^Nd/^144^Nd (fig. S2). Since the difference in mantle melting degree does not result in Nd isotope fractionation, it cannot be the primary cause for these correlated elemental variations.

An alternative explanation involves sub-arc mantle heterogeneity, suggesting that the enriched elemental and isotopic features in continental arc magmas may be attributed to an enriched ambient mantle similar to the EM-1 (enriched mantle 1) mantle end-member (*33*, *34*). In addition to highly enriched Sr-Nd isotopic features (high ^87^Sr/^86^Sr and low ^143^Nd/^144^Nd), the EM-1 mantle is characterized by distinctly low ^206^Pb/^204^Pb and ^207^Pb/^204^Pb values (*36*). Although Pb is highly mobile in slab-derived fluids and melts (*37*), the overprinting of mantle Pb isotopes by slab-derived Pb signature should be less pronounced in an Pb-rich enriched mantle. Consequently, if an EM-1–like mantle was to dominate magma compositions in continental arcs, then magmas with low ^143^Nd/^144^Nd should exhibit Pb isotopic signatures trending toward the EM-1 mantle; however, these variations are not widely observed in arc basalts (fig. S3). Therefore, the correlations of K_2_O and other incompatible elements with ^143^Nd/^144^Nd in primitive arc basalts are best explained by the involvement of slab-derived components, even for those conventionally “conservative” HFSEs such as Nb and Zr (fig. S4) (*32*). In contrast, heavy rare-earth element (HREE) contents (e.g., Yb) show no correlation with ^143^Nd/^144^Nd values (fig. S2), showing that Yb in arc magmas is sourced primarily from the ambient mantle rather than from the slab materials (*30*). The lower Yb contents in arc basalts [~1 to 3 parts per million (ppm)] compared to MORBs (~2 to 4 ppm) suggest extensively higher degrees of mantle melting or more depleted mantle sources (fig. S2) (*30*). While variations in ambient mantle composition and melting degree exist, both between arcs and within individual arcs (*35*), the preceding evidence indicates that these factors are subordinate in driving magma compositional variations on a global scale. By applying a depleted MORB mantle (DMM) source (*38*) to partial melting modeling, the results show that 15 to 25 wt % melting can well reproduce the observed Yb variations in most arc basalts (fig. S2).

Having excluded above possibilities, variations in K_2_O, other incompatible elements, and Sr-Nd isotopes must be attributed to slab-derived components. Potential components include aqueous fluids released by the dehydration of sediment, AOC, and serpentinite, as well as hydrous melts derived from the sediment and AOC layers. Low-K_2_O intraoceanic arc basalts typically display elevated B/Nb ratios (up to ~50) compared to MORBs and high-K_2_O arc magmas (generally <1) (fig. S5). Given that boron is extremely enriched in serpentinite, the high B/Nb ratios, along with the heavy boron isotopes [δ^11^B up to ~15 per mil (‰) (*19*) compared to −7.1‰ in MORB (*39*)] in low-K_2_O arc magmas, may therefore indicate substantial serpentinite-derived fluid modification (*19*). However, aqueous fluids, not only from the serpentinite layer, preferentially mobilize LILEs, leaving REEs and HFSEs largely immobile (*37*). The addition of aqueous fluids alone is therefore insufficient to explain the concurrent enrichment of REEs and HFSEs with K_2_O in arc basalts. Alternatively, these fluids can induce partial melting of the subducted sediment and AOC, producing hydrous melts that migrate into the mantle wedge (*11*, *13*). These melts can effectively transport LILEs and, crucially, exhibit mobilities for REEs and HFSEs that are approximately two orders of magnitude higher than those in aqueous fluids (*37*). For instance, at sub-arc depths, the melt-solid partition coefficient for Nb ($D_{\text{Nb}}^{\text{melt}/\text{solid}}$) lies between 1 and 10, whereas $D_{\text{Nb}}^{\text{fluid}/\text{solid}}$ is less than 0.1 (*37*). This enhanced mobility suggests that hydrous melts can be the ideal component to dominate the compositional shifts from low- to high-K_2_O arc magmas. Another proposed mechanism, the physical mixing of slab lithologies into the mantle wedge via mélange diapirs (*14*, *15*), is unlikely to be the predominant mass transfer agent. Although its geochemical signature has been identified in some arc segments (*40*), this process requires specific conditions that are not universally met (*41*).

**Fig. 4.**
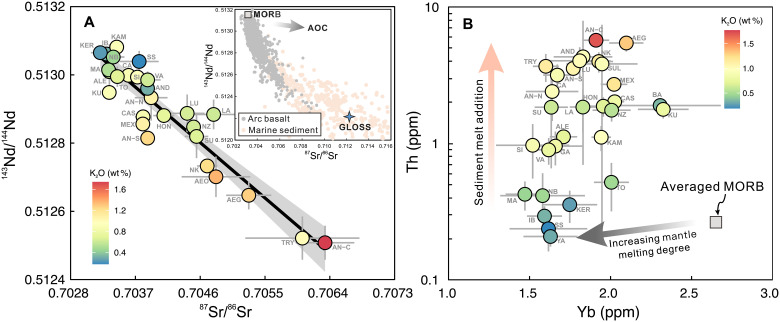
Identification of sediment melt contribution in sub-arc mantle based on primitive arc basalt isotopic and elemental features. Increasing magma K_2_O content (the color scale) correlates with more enriched radioactive Sr-Nd isotopes (**A**) and higher Th content (**B**), indicating a progressively greater sediment melt contribution from low- to high-K_2_O magmas. Conversely, the nearly constant Yb content with increasing K_2_O shows that the magma K_2_O variation is not controlled by the differences in mantle melting and/or ambient mantle compositions. GLOSS represents the updated “globally averaged subducted sediment composition” from Plank (*45*). The AOC Sr-Nd isotopic variation trend is from (*46*, *47*).

As the uppermost layer, the sediment veneer is particularly prone to melting due to higher slab surface temperatures (*42*, *43*). Furthermore, it serves as the primary host for K_2_O and most incompatible elements and exhibits enriched Sr-Nd isotopic signatures (Fig. 4A) (*44*, *45*). Collectively, these factors point to sediment melt as the dominant driver of compositional variability in arc basalts. In contrast, AOC-derived melts generally contain low-K_2_O contents and are characterized by elevated ^87^Sr/^86^Sr but MORB-like ^143^Nd/^144^Nd values (Fig. 4A) (*46*, *47*). Consequently, contributions from AOC melts are insufficient to explain the broad covariations observed between Sr-Nd isotopes, K_2_O, and other incompatible elements.

**Fig. 5.**
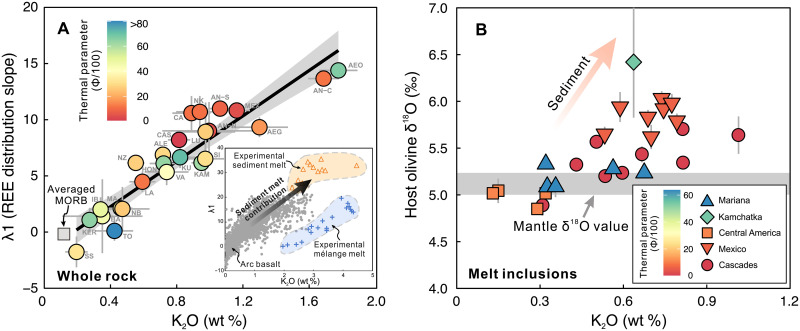
Relationship between sediment melt contribution indexes and slab subduction geotherm. Sediment melts are characterized by steep LREE/HREE distribution slopes (high λ1) (**A**) and elevated δ^18^O value (**B**). The positive correlations of whole-rock λ1 and olivine δ^18^O value with magma K_2_O content therefore indicate sediment melt contribution to K_2_O content variation. Mélange melts (*16*), by contrast, are insufficient to account for the magma λ1 variation with K_2_O content (A). The color scale is the thermal parameter (Φ) that reflects slab temperature, where low Φ values usually correspond to high slab temperatures (*113*). High-K_2_O magmas generally have low Φ values, showing that the sediment contribution is more pronounced in warm-to-hot subduction zones. The definition and calculation of λ1 follow O’Neill (*114*). Data in (A) are from the compiled Geochemistry of Rocks of the Oceans and Continents (GEOROC) whole-rock dataset (data S1), and those in (B) are from the olivine-hosted melt inclusions (data S3) (*101*).

The dominant role of sediment melt is further supported by Th abundances and Th/Yb ratios in primitive arc basalts. The Nb/Yb versus Th/Yb “Pearce diagram” (*32*) reveals that arc basalts show systematically elevation of Th/Yb ratios (i.e., Th excess) relative to the MORB array (fig. S4). Furthermore, high-K_2_O continental arc magmas exhibit notably higher Th abundances (~10- to 100-fold enrichment) and Th/Yb ratios than low-K_2_O intraoceanic arc magmas (Fig. 4B and fig. S4). Given that Th is strongly enriched in sediment melt (Fig. 2) (*48*), the observed Th excess in arc magmas implies a pervasive contribution from this component to the mantle source (Fig. 4B) (*13*), with the input being markedly more pronounced in continental arcs. As K_2_O and Th increase, Sr-Nd isotopic compositions trend toward the signatures of marine sediments (Fig. 4A), consistent with a progressively increasing sediment melt flux from low-K_2_O intraoceanic to high-K_2_O continental arc settings. Furthermore, experimental results show that sediment melts are characterized by elevated LREE/HREE ratios (*48*, *49*), represented by a steep REE distribution slope (λ1) (Fig. 5A). Global arc basalts show an increasing λ1 value with increasing K_2_O content, trending toward the high λ1 values observed in experimental sediment partial melts (Fig. 5A). Last, olivine-hosted, near-primary (MgO > 6 wt %) melt inclusions with high K_2_O also have host olivine δ^18^O values greater than the typical mantle value (Fig. 5B), confirming the involvement of high δ^18^O sediment in their source (*50*). These multiple lines of evidence consistently demonstrate the primary role of sediment melt in controlling arc basalt compositional variations. Continental arc magmas generally show stronger sediment melt signals, with higher K_2_O, LILE, Th, U, HFSE, and LREE contents and more enriched radiogenic Sr-Nd isotopes (Figs. 2 to 5). Partial melting calculations show that adding 1 to 10 wt % sediment melt to the mantle can well reproduce the trace element compositional ranges of global arc basalts (Fig. 2). Although other components such as AOC-derived melts, serpentinite-derived fluids, and mélange diapirs may also contribute, most geochemical fingerprints appear to be overprinted by the dominant sediment melt signature on the global scale.

A major control on the contribution of sediment melt is the subduction zone’s thermal structure. Most continental arcs, situated along the eastern Pacific rim, have higher slab temperatures than most intraoceanic arcs. Under these hotter conditions, sediments experienced higher degrees of partial melting, increasing the influx of sediment melt into the mantle wedge. Experiments also show that the concentrations of K_2_O and other incompatible elements in sediment melts increase with temperature (*48*, *51*). Consequently, both the sediment melt influx and its K_2_O content are elevated in hotter subduction zones, ultimately enhancing the K_2_O contents of arc magmas. This pattern is even evident within a single system such as the Vanuatu arc, where the Aoba volcano exhibits distinctly high-K_2_O, LREE, and Th contents, attributable to the subduction of the hot d’Entrecasteaux ridge (fig. S6) (*52*). The role of slab temperature is supported by the near-primary melt inclusion H_2_O/K_2_O and H_2_O/Ce thermometers (data S3) (*51*, *53*), where melt K_2_O contents show negative correlations with H_2_O/K_2_O and H_2_O/Ce ratios, implying higher slab surface temperatures beneath the high-K_2_O volcanic arcs (fig. S7).

However, the relationship between magma K_2_O content and slab temperature is not absolute, as other factors also play a role. Low H_2_O/K_2_O and H_2_O/Ce ratios, indicating high temperatures, are found across a wide range of K_2_O values (fig. S7). In the Cascades arc, where the slab is hot, relatively low-K_2_O contents may result from dilution by AOC-derived melts, as the mafic crust may undergo high degrees of partial melting (*54*). Conversely, magmas from the Aeolian arc in the Mediterranean region exhibit high K_2_O, low H_2_O/K_2_O, and H_2_O/Ce ratios despite only a moderate slab temperature (fig. S7). This arc is characterized by thick subducted sediment piles (*43*), suggesting that a high sediment input can raise the K_2_O flux into the mantle wedge independently of slab temperature.

### Role of different sediment types in the source of arc basalts

Although the globally averaged subducted sediment composition (GLOSS) can broadly explain global arc basalt compositional variations (Figs. 2, 4, and 5), marine sediment columns from oceanic drilling projects reveal substantial variability in sediment lithologies and compositions among different arcs (*45*, *55*). The incorporation of distinct sediment types into the sub-arc mantle may exert varying influences on both the compositional and *f*o_2_ variations in arc basalts. For example, Fe-Mn nodules, which are rich in highly oxidized Fe^3+^ and Mn^4+^, could enhance the oxidation of the sub-arc mantle upon subduction (*56*). Conversely, the subduction of organic carbon-rich sediments (e.g., black shale), which contain abundant reduced components, could suppress the mantle oxidation (*27*).

The U/K_2_O ratio serves as an effective geochemical tracer for differentiating the contribution of various sediment types to arc basalts. Uranium and potassium are highly incompatible in mantle minerals and exhibit similar geochemical behaviors during slab and mantle melting processes (*57*). This similarity suggests that the U/K_2_O ratio in arc basalts is primarily inherited from slab components and remains largely unaffected by slab melting processes.

**Fig. 6.**
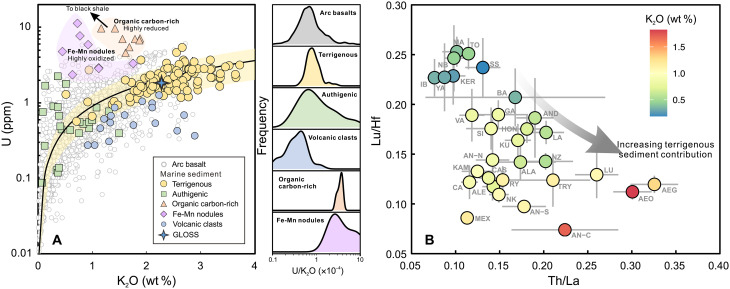
Identification of terrigenous sediment fingerprint from primitive arc basalt compositions. Terrigenous sediments exhibit high K_2_O and comparable U/K_2_O ratios to global arc basalts, serving as a high-K_2_O, high-U end-member for variations in arc basalt composition (**A**). The increase of magma K_2_O content also correlates with increasing Th/La and decreasing Lu/Hf ratios, showing increasing terrigenous sediment contribution in high-K_2_O magmas (**B**). Various types of sediment compositions in (A) are from Vervoort *et al.* (*58*).

Despite wide variations in K_2_O contents, global arc basalts maintain a relatively constant U/K_2_O ratio of (0.91 ± 0.05) × 10^−4^ (Fig. 6A). Among various sediment types, terrigenous sediments exhibit high-K_2_O contents and a U/K_2_O ratio [(0.90 ± 0.08) × 10^−4^] that closely matches that of arc basalts. This positions terrigenous sediment as the high-K_2_O, high-U end-member for the K_2_O-U variations observed in arc basalts (Fig. 6A). Other sediment types, in contrast, tend to have either insufficient K_2_O or excessive U to account for the observed compositional range.

The prominent role of terrigenous sediments is further supported by magma Lu/Hf and Th/La ratios. Terrigenous sediments typically exhibit low Lu/Hf ratios due to the presence of abundant detrital zircons that enriched in Hf (*58*), and they are also characterized by relatively high Th/La ratios (*59*, *60*). Consequently, the incorporation of terrigenous sediment melt into the mantle source should lead to decreased Lu/Hf and increased Th/La ratios in the resulting magmas. These trends are evident in global arc basalt data, which show decreasing Lu/Hf and increasing Th/La with rising K_2_O content (Fig. 6B). This evidence strongly suggests that the increase in K_2_O from intraoceanic to continental arc magmas is primarily driven by an enhanced contribution from terrigenous sediment melt. This conclusion aligns with observations from marine sediment columns. In most continental arcs, the near-trench sediment sequences are thick and dominated by terrigenous turbidites. In contrast, the sediment columns in intraoceanic arcs are generally thinner, and the terrigenous component is diluted by other types of sediments, such as authigenic and volcanic sediments (*45*, *55*). Moreover, terrigenous sediment represents the dominant component (>75%) in GLOSS (*55*), underscoring its preeminent role in driving variations in global arc basalt compositions. In addition to seafloor sediments, subduction erosion delivers substantial amounts of upper-plate terrigenous material from the fore-arc to sub-arc depths in continental margins, further contributing to the enriched compositions of arc magmas (*61*).

### Contribution of sediment melt to sub-arc mantle redox states

The addition of terrigenous-dominated sediment melts not only modifies the geochemical characteristics of arc basalts but may also fundamentally influence the redox state of the sub-arc mantle. The whole-rock V/Sc, V/Yb, and V/Ti ratios are widely used proxies to assess the *f*o_2_ of a magma’s mantle source (*3*, *62*). V is a multivalent element (ranging from V^2+^ to V^5+^), and its compatibility during mantle melting decreases as increasing *f*o_2_ drives it to higher valence states. In contrast, Sc, Yb, and Ti are redox-insensitive elements that behave similarly to V during melting, rendering the V/Sc, V/Yb, and V/Ti ratios sensitive indicators of redox conditions. However, recent modeling demonstrates that V/Yb and V/Ti ratios are also substantially affected by the degree of melting and source composition (*63*). Consequently, V/Sc is considered the most robust indicator of primary mantle *f*o_2_.

On the basis of the comparable V/Sc ratios between arc basalts and MORBs (fig. S8), Lee *et al.* (*62*) proposed that the sub-arc mantle is similarly reduced to the MORB mantle [~FMQ (fayalite-magnetite-quartz) − 1 to ~FMQ + 0.5]. However, subsequent experimental studies have revealed that variables such as temperature and melt composition also substantially affect V and Sc partitioning. Specifically, the relatively higher melting temperatures associated with MORB formation would result in lower *f*o_2_ values at similar V/Sc ratios (*3*). On the basis of a newly calibrated V/Sc-*f*o_2_ empirical model (*3*), the calculated *f*o_2_ values in sub-arc mantle are ~1 log unit higher than the MORB mantle despite the similar magma V/Sc ratios (*3*, *63*) (fig. S8).

**Fig. 7.**
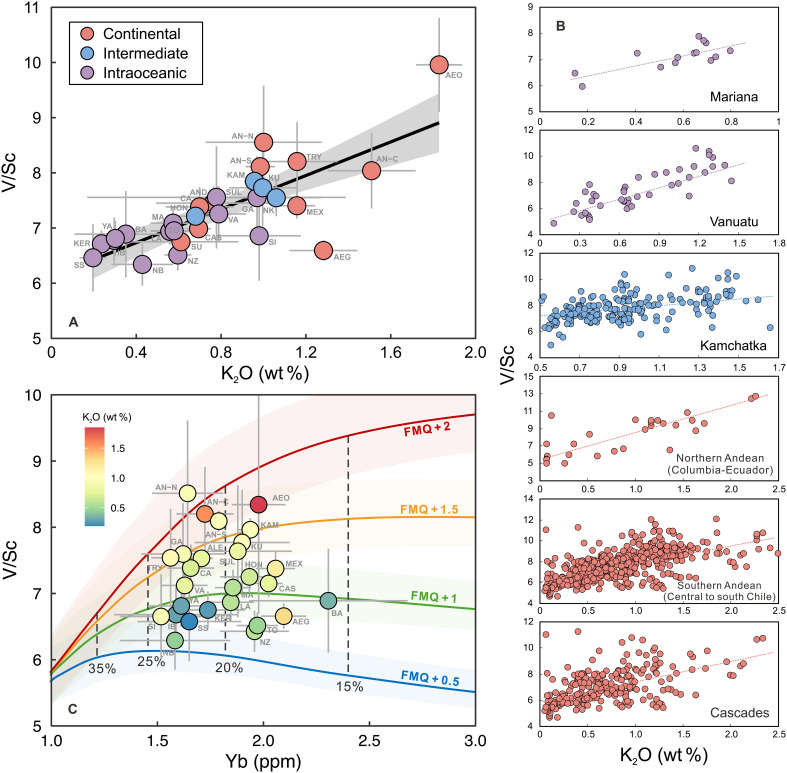
Variations in arc basalt V/Sc ratios with sediment melt contribution in their source. (**A** and **B**) illustrate the V/Sc variations in relation to K_2_O content from a global perspective (A) and six representative arcs (B). (**C**) Effect of mantle melting degree on melt V/Sc, with Yb as an indicator for mantle melting degree. Global arc basalts have narrow variations in Yb contents, showing that the V/Sc variations are caused by melting at different *f*o_2_ conditions, rather the result of varying mantle melting degree. Shaded areas in (C) indicate the errors of *f*o_2_ estimation, which were calculated on the basis of uncertainties of 7% for V and 13% for Sc in DMM (*64*). Detailed modeling methods for V/Sc variation with *f*o_2_ and melting degree are provided in Materials and Methods, with the modeled results available in data S4.

Although their averaged V/Sc ratios are similar, arc basalts display a much broader range of V/Sc values than MORBs (fig. S8) and show a positive correlation with K_2_O contents, both on a global scale (Fig. 7A) and within individual arcs (Fig. 7B). This trend suggests that the mantle source *f*o_2_ may increase with a greater contribution from sediment melt, a conclusion further corroborated by the positive correlation between estimated sediment melt influx and magma V/Sc ratios (fig. S9). These correlations also challenge the hypothesis that serpentinite-derived fluids are the primary oxidizing agents (*19*, *20*). If serpentinite dehydration was the dominant source of oxidizing components, then low-K_2_O intraoceanic arc magmas, which bear clear geochemical signatures of serpentinite-fluid involvement (fig. S5), should exhibit elevated V/Sc ratios and correspondingly high *f*o_2_ (*19*). However, the observed trends demonstrate the opposite (Fig. 7).

Nevertheless, other factors—such as variations in mantle melting degree, mantle source heterogeneity, and the direct addition of V and Sc from sediments—could also influence V/Sc ratios. To validate the oxidation role of sediment melt, these factors must be carefully evaluated.

Here, we modeled V/Sc variations during the partial melting of a sub-arc mantle peridotite, using Yb content as a proxy for the degree of mantle melting (Fig. 7C and data S4). DMM values for V (79 ppm), Sc (16.3 ppm) (*64*), and Yb (0.365 ppm) (*38*) were used for the modeling. The model results show that melt V/Sc ratios vary minimally at lower *f*o_2_ conditions (FMQ + 0.5 to FMQ + 1). At higher *f*o_2_ conditions (FMQ + 1.5 to FMQ + 2), the V/Sc ratio decreases as melting degree increases (i.e., lower Yb). In global arc basalts, however, Yb content does not correlate with the V/Sc ratio, indicating that the degree of mantle melting is not the primary driver of V/Sc variations. Regarding mantle heterogeneity, the compositional uncertainties in DMM (from depleted DMM to enriched DMM) V and Sc are ~7 and 13%, respectively (*64*). Even when considered, the resulting uncertainty in the calculated melt V/Sc is insufficient to explain the observed variation in average magma V/Sc ratios from ~6 to ~8.5 among different arcs (Fig. 7C).

Another alternative is the direct incorporation of high-K_2_O, high-V/Sc sediment melts. Averaged subducted sediment (GLOSS) contains 116-ppm V and 15-ppm Sc (*45*). During sediment melting at sub-arc depths, the bulk partition coefficient of V transitions from compatible to slightly incompatible ($D_{V}^{\text{Bulk}}$ = ~0.5 to 1.8) as *f*o_2_ increases from ~FMQ − 1 to ~FMQ + 4 (*49*, *65*), while Sc remains slightly compatible ($D_{\text{Sc}}^{\text{Bulk}}$ = ~1.4) (data S5). As a result, V and Sc contents in the resulting sediment melt do not show notable enrichment or depletion, although the V content and V/Sc ratio do increase with *f*o_2_ (fig. S10). Compared to the depleted mantle V (79 ppm) and Sc (16.3 ppm) contents (*64*), sediment melts have comparable to slightly higher V (67 to 167 ppm) and slightly lower Sc (10 to 14 ppm). By adding 10 wt % of an oxidized sediment melt to the DMM, the resulting magma V/Sc ratio increases by only ~1 unit compared to a pure DMM melt at the same *f*o_2_ condition (fig. S8). Given that sediment melt fractions are generally less than 10% (Fig. 2), the V/Sc variation caused by direct sediment melt addition is also insufficient to explain the full range observed in arc basalts (fig. S8). This inference is reinforced by the lack of correlation between the V/Sc ratio of subducted sediments at different arcs and the V/Sc ratio of the corresponding arc magmas (fig. S11) (*45*, *55*), confirming that direct sediment input does not control the magma V/Sc variations. Therefore, the V/Sc variations in primitive arc basalts are reflective of variations in their source *f*o_2_. The low-K_2_O, low-V/Sc intraoceanic arc magmas are derived from less oxidized sub-arc mantle sources with *f*o_2_ as low as ~FMQ + 0.5. In contrast, the high-K_2_O, high-V/Sc continental arc magmas originate from more oxidized sources with *f*o_2_ reaching up to ~FMQ + 2 (Fig. 7C).

While the robustness of whole-rock V/Sc redox proxy has been rigorously assessed, the model relies on assumptions about mantle composition, mineral assemblages, and melting reactions. Hence, the results necessitate further validation through other redox proxies. Olivine-hosted melt inclusions offer an alternative method for determining *f*o_2_ values, using either the Fe^3+^/∑Fe ratio of the melt or the V partitioning between the host olivine and the melt (*66*). However, melt inclusions can be trapped at various stages of magma differentiation, meaning that their *f*o_2_ may not represent the mantle source (*67*). Furthermore, postentrapment processes such as H_2_O diffusion and magma degassing can notably alter the Fe^3+^/∑Fe ratio, potentially biasing *f*o_2_ estimates (*68*, *69*).

**Fig. 8.**
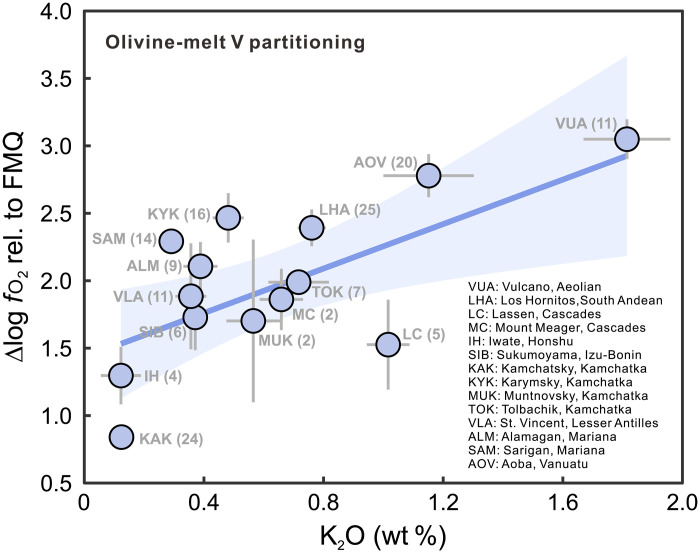
Variations of *f*o_2_ values recorded melt inclusions with melt K_2_O contents. The *f*o_2_ values are calculated by host olivine-melt inclusions V partitioning. Detailed calculation information is provided in Materials and Methods and data S3.

To mitigate these issues, we selected only near-primary melt inclusions (MgO > 6 wt %) and relied primarily on results obtained from V partitioning. On the basis of data from 14 volcanoes across nine arcs, the calculated *f*o_2_ values increase from ~FMQ + 1 to ~FMQ + 3 as the inclusion K_2_O content rises from ~0.1 to ~1.8 wt % (Fig. 8). The clear trend from V partitioning aligns perfectly with that from whole-rock V/Sc ratios, confirming that the incorporation of sediment melt increases both the K_2_O content and the degree of oxidation in arc magmas. This conclusion is further supported by positive correlations of K_2_O content and the minimum *f*o_2_ estimated from inclusion sulfur content (*70*) (fig. S12). In addition, the host olivine-melt Mg-Fe^T^ (total iron) exchange coefficients (${\mathrm{KD}}_{{\text{Fe}}^{T}-\text{Mg}}^{\text{Ol}-\text{melt}}$) decreases with increasing *f*o_2_ (*71*), and, thus, the observed negative correlation between inclusion K_2_O content and the ${\mathrm{KD}}_{{\text{Fe}}^{T}-\text{Mg}}^{\text{Ol}-\text{melt}}$ value further indicates elevated *f*o_2_ values in high-K_2_O magmas (fig. S13).

Although both whole-rock V/Sc and olivine-melt V partitioning reveal a consistent increase in *f*o_2_ with magma K_2_O contents, the absolute *f*o_2_ values calculated from V partitioning are systematically higher by ~1 log unit. This discrepancy possibly reflects further oxidation during magma ascent (*8*, *72*). Although the inclusions are near primary (MgO > 6 wt %), they record redox conditions at the time of olivine crystallization, typically at crustal pressures (*73*). As hydrous magma ascends through the mantle wedge, it can be oxidized by H_2_O dissociation and H_2_ incorporation into the surrounding mantle (*72*). This process would systematically shift the *f*o_2_ recorded by the inclusions to higher values, potentially explaining the offset from the mantle source *f*o_2_ recorded by the whole-rock V/Sc proxy.

### Mechanism of oxidation in the sub-arc mantle

The redox state of the sub-arc mantle is primarily controlled by the influx of multivalent, redox-sensitive elements such as carbon, sulfur, and iron from slab components (*4*). Carbon is predominantly stored in the sediment veneer, with two-thirds of the total subducted carbon (up to ~60 million tonnes/year) transported as both reduced organic carbon (C^0^) and oxidized carbonate (C^4+^) forms (*28*, *74*). However, reduced organic carbon has low solubility in slab fluids and melts, limiting its migration into the mantle wedge and thus minimizing its influence on the mantle’s redox state (*75*). This conclusion is reinforced by the observation that continental arcs along the eastern Pacific generally received greater organic carbon input than intraoceanic arcs in the western Pacific (*74*), yet their sub-arc mantle *f*o_2_ values are typically higher, not lower. Furthermore, the distinctively high-U contents and U/K_2_O ratios of organic carbon-rich sediments are not reflected in arc basalts (Fig. 6A), arguing against their substantial contribution. In contrast, while carbonate can be effectively mobilized by slab fluids and melts, its addition is unlikely to cause notable oxidation. The redox state of the unmodified sub-arc mantle lies on or slightly above the graphite-CO_2_ buffer (~FMQ − 1), where carbonate is a stable species (*76*). Therefore, injecting more carbonate does little to change the overall *f*o_2_. Although experimental studies indicate that carbonate can oxidize Fe^2+^ to Fe^3+^ in silicates, raising *f*o_2_ (*77*, *78*), there is no evidence that these redox reactions occur at sub-arc depths.

With respect to sulfur, sulfide (S^2−^) is the stable species in the ambient mantle, implying that the addition of slab-derived S^2−^ has little influence on the sub-arc mantle’s *f*o_2_ (*4*). Sulfate (S^6+^), however, is highly soluble in slab fluids and/or melts and has a strong oxidation capacity, making it a potential driver of *f*o_2_ variations (*79*–*81*). Thermodynamic models suggest that sulfur in the AOC and serpentinite layers can be progressively oxidized from S^2−^ to S^6+^ at depth through reactions with Fe^3+^. The resulting S^6+^ could then dissolve into aqueous fluids, oxidizing the sub-arc mantle (*18*). However, this aqueous fluid S^6+^ oxidation model fails to explain the observed correlation between magma K_2_O content and *f*o_2_, as magma K_2_O is controlled primarily by sediment melt, not fluids from the AOC or serpentinite. In addition, the conversion from reduced to oxidized sulfur would release chalcophile elements, such as Cu, into the sub-arc mantle, potentially resulting in elevated Cu concentrations in primary arc magmas. However, Cu concentrations in oxidized arc basalts are no higher than in reduced MORBs (*5*, *82*), suggesting that the transport of S^6+^ via this fluid pathway is limited (*21*, *23*). This is consistent with observations from natural samples, where sulfide remains the dominant sulfur species in dehydration-related veins within subducted eclogites (*21*).

The limited oxidizing capacity of slab fluids suggests that the *f*o_2_ of the subducted AOC and serpentinite may not be sufficient to account for the oxidized nature of the sub-arc mantle (*21*). In contrast, marine sediments, particularly those deposited since the Great Oxidation Event, are highly oxidized because of prolonged exposure to oxygenated seawater and continental weathering (*26*, *83*). This environment fosters the formation of abundant evaporitic sulfate (*84*) and Fe^3+^-bearing phases (e.g., hematite, magnetite, and goethite) (*24*). While sediments also contain reduced organic carbon, its preservation requires specific conditions (e.g., high productivity and rapid burial) and is more reflective of localized phenomena (*85*).

**Fig. 9.**
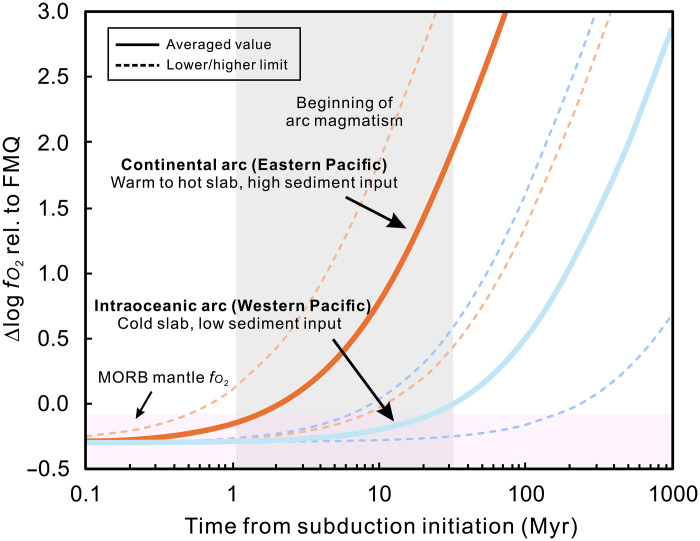
Difference of sub-arc mantle oxidation efficiency caused by sediment melt in different arcs. The calculations are performed on the basis of the RB concept proposed in Evans (*87*). Fe^3+^ and S^6+^ are treated as the primary oxidizing agent in sediment melt. Arc magmatism usually occurs since 1.8 to 52 million years (Myr) (10^6^ years) after the subduction initiation (*115*). For detailed information, see Materials and Methods and table S1.

At sub-arc depths, the partial melting of these highly oxidized sediments releases S^6+^ from sulfate and Fe^3+^ from iron oxides into the melt. Experimental data show that the concentrations of S^6+^ (at anhydrite saturation) and Fe^3+^ (at hematite-magnetite saturation) in sediment melt increase notably from ~0.01 to ~0.04 wt % (*80*) and <0.1 to ~1 wt % (*86*), with rising temperature at sub-arc depths. By applying a redox budget (RB) calculation proposed by Evans (*87*), we find that the incorporation of these S^6+^- and Fe^3+^-rich melts, particularly in warmer subduction zones, can effectively oxidize the sub-arc mantle on timescales relevant to arc magma formation (Fig. 9). This mechanism elegantly explains the observed link between magma composition and redox state: Hotter subduction zones produce higher degrees of sediment melting, which simultaneously delivers more K_2_O, Fe^3+^, and S^6+^ to the mantle wedge, resulting in the high-K_2_O, highly oxidized arc basalts typical of continental arcs. Conversely, in colder intraoceanic arcs, lower degrees of sediment melting lead to a smaller influx of these components, producing low-K_2_O, less oxidized magmas.

While sediment melt is a highly efficient and primary oxidizing agent, it may not be the sole mechanism. Although the direct dissolution of oxidized sulfur in aqueous fluids appears insufficient to oxidize the sub-arc mantle within a reasonable timescale, a secondary process may also contribute. Approximately 6.4% of total subducted sulfur is lost to these fluids, mainly in the form of H_2_S and HS^−^ (*21*). As these fluids ascend from deeper within the slab, they may be oxidized upon passing through the highly oxidized sediment veneer at the slab surface (*24*). This interaction would also generate S^6+^-bearing fluids, providing an additional pathway for oxidizing the sub-arc mantle.

### Implications for the origin of porphyry deposits

Compared to intraoceanic arcs, many continental arcs are distinguished not only by high-K_2_O, high-*f*o_2_ arc lavas but also by their exceptional metallogenetic potential for porphyry Cu-Au deposits (*7*, *88*). The spatial association is notable: Most giant porphyry deposits are associated with high-K_2_O arc lavas (Fig. 1). This association extends to postcollisional settings, where porphyry deposits are typically linked to K-rich magmas derived from metasomatized mantle that has been influenced by continental crust materials (*89*–*91*). Recent studies on Tethyan magmas have shown that the subduction of terrigenous sediments during continental collision caused mantle oxidation, thereby facilitating porphyry deposit formation in postcollisional environments (*91*, *92*). The strong spatial correlation between giant porphyry deposits and high-K_2_O arc magmas suggests that oxidized sediment melts also play a crucial role in porphyry deposit formation within oceanic subduction settings (*90*).

The most straightforward mechanism by which sediment melts might contribute to Cu-Au mineralization would be through the direct delivery of these metals to the magma source (*93*, *94*). However, global arc basalt data show that high-K_2_O continental arc lavas do not exhibit elevated Cu content compared to low-K_2_O lavas (fig. S14). More tellingly, Cu and Au concentrations in unmodified melt inclusions from K-rich mafic rocks do not differ notably from those in K-poor rocks (*95*), suggesting that Cu-Au enrichment in the mantle source is not a prerequisite for successful mineralization. Instead, the high *f*o_2_ in the source, largely imparted by oxidized sediment melts, appears to be the key factor regulating Cu-Au enrichment during magma differentiation (*90*). In porphyry deposit–barren intraoceanic arcs, the relatively low *f*o_2_ values (~FMQ + 0.5) cannot suppress sulfide saturation during magma differentiation. The resulting sulfide precipitation sequesters chalcophile elements such as Cu and Au in deep cumulate rocks, preventing their concentrations in evolved melts (*5*, *96*). In continental arcs, however, the high *f*o_2_ (~FMQ + 1.5) of the primitive magmas, along with further oxidation during magma ascent (*72*), effectively inhibits sulfide saturation. This maintains Cu-Au in the melt phase through much of the differentiation process, allowing these metals to concentrate in evolved magmas and ultimately creating favorable conditions for the exsolution of ore-forming fluids during the final stages of crystallization (*96*).

## MATERIALS AND METHODS

### Data compilation

Global igneous rock data from convergent margins were downloaded from the Geochemistry of Rocks of the Oceans and Continents (GEOROC) database (https://georoc.mpch-mainz.gwdg.de/georoc/). The raw data were imported into GeoMapApp (www.geomapapp.org/) to select samples located at Cenozoic volcanic arcs. Samples located at Cenozoic arcs but have non-Cenozoic ages were also removed. To obtain near-primary magmatic samples from sub-arc mantle melting, we only kept volcanic samples with MgO = 6 to 12 wt %, Mg# = 60 to 72, SiO_2_ = 45 to 54 wt % and loss on ignition < 5 wt %. These criteria minimize the influence of magma differentiation and mixing, crustal assimilation, crystal cumulation, and surface alteration. In addition, we removed samples with K_2_O > 3 wt % to ensure better *f*o_2_ comparison using the V/Sc redox proxy. The source lithologies of these extremely high-K_2_O samples are complex (e.g., phlogopite pyroxenite and phlogopite peridotite) compared to the simple four-phase mantle lithologies (*97*–*99*), which alter the melt-bulk residue V and Sc partitioning during mantle melting and thus obscures the redox conditions obtained by the V/Sc proxy. Accordingly, we collected a total of 4525 records from 33 arcs, of which about half contain trace element data and about a quarter include Sr-Nd isotopic data (data S1).

MORB data were obtained from a precompiled dataset by Gale *et al.* (*100*) (data S2). To enable direct comparison with those primary arc basalts, we only kept data with MgO = 6 to 12 wt % and Mg# = 60 to 72. According to these criteria, 914 records on MORB data were obtained.

Olivine-hosted melt inclusion data from arc settings, with olivine-melt V partition coefficients available, were compiled from literature (data S3). In addition, we collected melt inclusion data where host olivine O isotope was reported, and melt H_2_O/Ce and H_2_O/K_2_O ratios can be calculated. The olivine O isotope data are from a precompiled dataset by Ruscitto *et al.* (*101*). For the melt inclusion data, if we apply the same data filtering criteria as for the whole-rock samples to select near-primary melts, the resulting dataset would be too limited. We therefore used MgO > 6 wt % and Mg# > 50 as the criteria to select near-primary melt inclusions. Records with abnormally high olivine-melt Fe^T^-Mg exchange coefficient (${\mathrm{KD}}_{{\text{Fe}}^{T}-\text{Mg}}^{\text{Ol}-\text{melt}}$ > 0.5) were also removed. The data screening and following calculation processes are based on the postentrapment-corrected melt compositions.

### Modeling of V/Sc variations during sediment melting and mantle melting processes

To evaluate the melt V and Sc contents during sediment melting and further assess its influence on mantle V and Sc contents, we applied a batch melting model

$$c_{i}=\frac{c_{0}}{F+\left(1-F\right)\times D}$$ (1)

where $c_{i}$ and $c_{0}$ are the V or Sc contents in the partial melt and bulk sediment, respectively. F is the melting degree, and D is the bulk partition coefficient of V or Sc between the residue and the melt.

For $c_{0}$, we used the V (116 ppm) and Sc (15 ppm) contents in GLOSS (*45*). The calculation of D requires residual phase proportions and mineral-melt V and Sc partition coefficients. Phase proportions were taken from the fluid-saturated sediment melting experiments of Mann and Schmidt (*102*) at 3 GPa and 800° to 1200°C, in which the major residual phases include clinopyroxene, garnet, rutile, phengite, coesite, and kyanite. All the phases are typical residual phases during sediment melting (*49*, *51*). Among these phases, V and Sc are compatible in clinopyroxene, garnet, rutile, and phengite across wide *f*o_2_ conditions (FMQ − 1 to FMQ + 3.7) (*65*, *103*), with V partition coefficients decreasing with increasing *f*o_2_ (*65*). Here, quantitative constraints on the *f*o_2_ dependence of phengite-melt V partition coefficient are lacking, and, thus, this value was assumed to be a constant at various *f*o_2_ conditions (*103*). Regarding coesite and kyanite, the V and Sc partitioning data are lacking, and, thus, the values were arbitrarily set to 0, considering that these two phases should be incompatible to V and Sc. These assumptions would slightly underestimate their contribution to bulk partition coefficients and thus results in a minor overestimation on melt V and Sc contents. On the basis of these parameters, the calculated bulk V partition coefficient ($D_{V}^{\text{Bulk}}$) decreases from 1.1 to 1.8 at FMQ − 1 to 0.5 to 0.9 at FMQ + 3.7, and the Sc partition coefficient has a limited range from 1.2 to 1.6 across the above *f*o_2_ range.

The modeling results show that both melt V and Sc contents increase with increasing melting degree, with the melt V/Sc ratio remaining nearly constant at any given *f*o_2_ condition (fig. S10). Compared to the initial GLOSS V content at 115 ppm, the melt V contents are depleted at FMQ − 1 but increase to match or exceed initial values (by up to 40 ppm) at relatively oxidized conditions (FMQ + 1.7 to FMQ + 3.7) (fig. S10). Melt Sc contents, which are independent of *f*o_2_, range from ~10 to 13.7 ppm, slightly below the GLOSS Sc value (15 ppm). As melt V content increases with *f*o_2_, while Sc remains stable, melt V/Sc ratios increase from ~6.5 at FMQ − 1 to ~13.6 at FMQ + 3.7 (fig. S10). Detailed partitioning parameters, data sources, and calculation outputs are provided in data S5.

Compared to the DMM V (79 ppm) and Sc (16.3 ppm) contents (*64*), the sediment melts do not have notably higher V and Sc contents. For less than 10% sediment melt incorporation into the mantle, its influence on mantle V content and V/Sc ratio are less than ~10 and ~ 5%, respectively (fig. S10). As sediment melt V and Sc contents increase with increasing temperature and melting degree, its influence on mantle V and V/Sc becomes slightly increased in warm-to-hot subduction zones.

For sub-arc and MORB mantle melting scenarios, a near-fractional melting model was applied, where detailed information about mantle mineral constitutions, melting reactions, and mineral-melt V and Sc partition coefficients (*D*_V_ and *D*_Sc_) under various *f*o_2_ conditions was described in Liu *et al.* (*63*). DMM V (79 ppm) and Sc (16.3 ppm) contents were applied for both sub-arc and MORB mantle melting scenarios, and the uncertainties are 7% for V and 13% for Sc (*64*), respectively. Yb is immobile during slab dehydration and melting (*30*), as, thus, it was used as a tracer for mantle melting degree on both scenarios. DMM Yb content of 0.365 ppm was applied (*38*), and the mantle mineral-melt Yb partition coefficients were from McKenzie and O’Nions (*104*).

Melting temperatures were set to initiate from 1150° and 1300°C for sub-arc and MORB mantle, respectively (*3*, *63*). As mantle mineral-melt *D*_V_/*D*_Sc_ decreases with increasing temperatures (*3*), melting at relatively high temperature would result in elevated melt V/Sc ratios at a constant *f*o_2_. This temperature effect explains why arc basalts and MORBs have similar V/Sc ratios but different *f*o_2_ values (fig. S8) (*3*). The calculation results show that arc basalts were primarily generated at *f*o_2_ conditions from <FMQ + 0.5 to >FMQ + 2 (fig. S8). In contrast, the V/Sc variation in MORBs can be well reproduced by melting of a relatively reduced mantle with *f*o_2_ of FMQ − 0.5 to FMQ + 0.5 (fig. S8).

To investigate the influence of oxidized sediment melt, a mixed mantle source of DMM + 10% sediment melt was applied to the sub-arc melting process. High (*F* = 34.6 wt %, 950°C) degree sediment melt formed at 3 GPa, and oxidized condition (FMQ + 3.7) was used as the end-member, under which conditions the sediment melt has the highest V and V/Sc ratios (fig. S10). Applying such a mixed source can therefore evaluate the maximum effect of sediment melt incorporation. Calculation results show that incorporating 10% sediment melt into the mantle source alters the resulting melt V/Sc ratio by less than 1 unit at a given *f*o_2_, insufficient to account for the full observed range of V/Sc values (~6 to ~10) in arc magmas (fig. S8). Full details of the modeling parameters and results are provided in data S4.

### Oxygen fugacity calculation from melt inclusion data

Melt inclusion–based *f*o_2_ values were determined using host olivine-melt V partitioning method and the empirical equation of Erdmann *et al.* (*105*)

$${fo}_{2}\left(\text{ΔFMQ}\right)=-2.8438-3.1960\;\times \;\text{log}D_{V}-0.8781\;\times \;\text{hNBO}/T$$ (2)

Here, $D_{V}$ is the partition coefficient between host olivine and melt inclusion, and hNBO/T is the hydrous-based nonbridging oxygen/tetrahedral cation ratio in the melt inclusion. The calculation of hNBO/T requires melt water content, and for data with missing water contents, the inclusion water content is assumed to be at 4 wt % (*106*). Detailed calculation of hNBO/T is listed in data S3, and the method can be found in Erdmann *et al.* (*105*).

To check the consistency of different methods, we also calculated the V partitioning based *f*o_2_ values using the empirical equation of Wang *et al.* (*3*). In their method, melt NBO/T value and melting temperature are two required variables, and the calculation of these variables are based on the methods of (*107*, *108*). The two methods show consistent results with the *f*o_2_ difference less than 0.5 log unit (fig. S15).

### RB calculation

The RB (${\dot{\text{RB}}}_{M}$) is an extensive variable that describes a geologic system’s oxidizing capacity, determined by the quantity of redox-sensitive elements present and their valence states. For the sub-arc mantle oxidation process, the ${\dot{\text{RB}}}_{M}$ of incorporated sediment melt can be expressed by

$${\dot{\text{RB}}}_{M}=\sum_{i}n_{i}\times v_{i}$$ (3)

Here, $n_{i}$ is the flux of redox-sensitive element *i* (C, S, or Fe) into the sub-arc mantle (in moles per year), and $v_{i}$ is the number of electrons required to take 1 mol of element *i* at a specific initial valence state to the sub-arc mantle reference valence state. The reference valence states of C, S, and Fe in sub-arc mantle are C^4+^, S^2−^, and Fe^2+^, respectively (*4*, *76*). In sediment melts, C^0^ is insoluble (*75*), and the ${\dot{\text{RB}}}_{M}$ of dissolved C^4+^ is zero. Therefore, sub-arc mantle oxidation by sediment melt is mainly caused by the incorporation of hypervalent S (S^6+^) and Fe^3+^ in the melt, in which the $v_{i}$ value is 8 mol for S^6+^ (S^2−^ to S^6+^) and 1 mol for Fe^3+^ (Fe^2+^ to Fe^3+^).

For per unit (in meters) arc length, the flux of sediment melt S^6+^ or Fe^3+^ into sub-arc mantle can be calculated by

$$n_{i}=\left(D_{\text{sed}}\times v_{\text{slab}}\times F_{\text{sed}}\times c_{i}\times \rho \right)/M_{i}$$ (4)

Here, $D_{\text{sed}}$ is the thickness of subducted sediment; $v_{\text{slab}}$ is the slab subduction velocity; $F_{\text{sed}}$ is the melting degree of subducted sediment at sub-arc depths; $c_{i}$ is the concentration of S^6+^ or Fe^3+^ in the sediment melt; ρ (2.7 × 10^6^ g/m^3^) is the density of subducted sediment; and $M_{i}$ is the molar weight of S^6+^ or Fe^3+^. The ranges of $D_{\text{sed}}$ and $F_{\text{sed}}$ were set to in 200 to 600 m and 10 to 30 wt % in relatively cold, intraoceanic subduction zones, and 300 to 1000 m and 20 to 40 wt % in warm-to-hot, continental subduction zones (*43*). The $v_{\text{slab}}$ was set to 0.04 to 0.07 m/year (*43*). S^6+^ and Fe^3+^ in the sediment melt were set to 0.01 wt % and 0.04 to 0.16 wt % in cold subduction zones and 0.04 wt % and 0.69 to 1.11 wt % in warm-to-hot subduction zones (*79*, *80*, *86*). These values represent the S^6+^ content at anhydrite saturation and Fe^3+^ at hematite-magnetite saturation. All the data used in the calculation, with the data source, are listed in table S1.

The calculation results show that the ${\dot{\text{RB}}}_{M}$ of S^6+^ varies from 54 to 850 mol/year per meter arc length in cold, intraoceanic subduction zones and from 648 to 7560 mol/year per meter in warm-to-hot, continental subduction zones. To further establish a link between the extensive variable ${\dot{\text{RB}}}_{M}$ and the intensive variable *f*o_2_, the following equations were applied (*76*)

$${\bar{\text{RB}}}_{M}={\bar{\text{RB}}}_{M,\text{init}}+\frac{{\dot{\text{RB}}}_{M}\times \Delta t}{M}$$ (5)

$${fo}_{2}\left(\text{ΔFMQ}\right)=4.63+1.57\times \text{ln}{\bar{\text{RB}}}_{M}$$ (6)

Here, ${\bar{\text{RB}}}_{M}$ is the specific RB of sediment melt in moles per kilogram, and ${\bar{\text{RB}}}_{M,\text{init}}$ is the specific RB of the unmodified sub-arc mantle. Δt represents the time taken for the system to reach a new steady state after the onset of subduction (in years). M is the mass of mantle wedge affected by slab sediment melt (see fig. S16 for details). Equation 6 was constructed on the basis of a mantle sulfur content of 100 ppm, comparable to the estimated mantle sulfur content (*109*). ${\bar{\text{RB}}}_{M,\text{init}}$ is calculated from Eq. 6 by assuming an unmodified sub-arc mantle *f*o_2_ at FMQ − 0.3 (*110*).

## Supplementary Materials

### The PDF file includes:

Supplementary Text Figs. S1 to S16 Table S1 Legends for data S1 to S6 References

## References

[1] E. Cottrell, S. K. Birner, M. Brounce, F. A. Davis, L. E. Waters, K. A. Kelley, “Oxygen fugacity across tectonic settings,” in *Magma Redox Geochemistry*, R. Moretti, D. R. Neuville, Eds. (Wiley, 2021), pp. 33–61.

[2] K. A. Kelley, E. Cottrell, Water and the oxidation state of subduction zone magmas. Science 325, 605–607 (2009). 19644118 10.1126/science.1174156

[3] J. Wang, X. Xiong, E. Takahashi, L. Zhang, L. Li, X. Liu, Oxidation state of arc mantle revealed by partitioning of V, Sc, and Ti between mantle minerals and basaltic melts. J. Geophys. Res. Solid Earth 124, 4617–4638 (2019).

[4] K. A. Evans, The redox budget of subduction zones. Earth Sci. Rev. 113, 11–32 (2012).

[5] J. P. Richards, The oxidation state, and sulfur and Cu contents of arc magmas: Implications for metallogeny. Lithos 233, 27–45 (2015).

[6] W. Sun, R.-f. Huang, H. Li, Y.-b. Hu, C.-c. Zhang, S.-j. Sun, L.-p. Zhang, X. Ding, C.-y. Li, R. E. Zartman, M.-x. Ling, Porphyry deposits and oxidized magmas. Ore Geol. Rev. 65, 97–131 (2015).

[7] C.-T. A. Lee, M. Tang, How to make porphyry copper deposits. Earth Planet. Sci. Lett. 529, 115868 (2020).

[8] M. Tang, M. Erdman, G. Eldridge, C.-T. A. Lee, The redox “filter” beneath magmatic orogens and the formation of continental crust. Sci. Adv. 4, eaar4444 (2018). 29774235 10.1126/sciadv.aar4444 PMC5955626

[9] I. J. Parkinson, R. J. Arculus, The redox state of subduction zones: Insights from arc-peridotites. Chem. Geol. 160, 409–423 (1999).

[10] S.-Y. Zhao, A. Y. Yang, C. H. Langmuir, T.-P. Zhao, Oxidized primary arc magmas: Constraints from Cu/Zr systematics in global arc volcanics. Sci. Adv. 8, eabk0718 (2022). 35319995 10.1126/sciadv.abk0718 PMC8942352

[11] T. Elliott, “Tracers of the slab,” in *Inside the Subduction Factory*, J. Eiler, Ed. (American Geophysical Union, 2003), pp. 23–45.

[12] H. Li, J. Hermann, L. Zhang, Melting of subducted slab dictates trace element recycling in global arcs. Sci. Adv. 8, eabh2166 (2022). 35020421 10.1126/sciadv.abh2166 PMC10954032

[13] S. J. Turner, C. H. Langmuir, Sediment and ocean crust both melt at subduction zones. Earth Planet. Sci. Lett. 584, 117424 (2022).

[14] E. A. Codillo, V. Le Roux, H. R. Marschall, Arc-like magmas generated by mélange-peridotite interaction in the mantle wedge. Nat. Commun. 9, 2864 (2018). 30030428 10.1038/s41467-018-05313-2 PMC6054672

[15] S. G. Nielsen, H. R. Marschall, Geochemical evidence for mélange melting in global arcs. Sci. Adv. 3, e1602402 (2017). 28435882 10.1126/sciadv.1602402 PMC5384804

[16] A. M. Cruz-Uribe, H. R. Marschall, G. A. Gaetani, V. Le Roux, Generation of alkaline magmas in subduction zones by partial melting of mélange diapirs—An experimental study. Geology 46, 343–346 (2018).

[17] L. M. Saper, M. Brounce, D. Woelki, R. Cao, G. Bromiley, Variable oxidizing capacity of slab-derived fluids: Insights from Fe and S speciation in glasses from the Troodos Ophiolite. Earth Planet. Sci. Lett. 627, 118560 (2024).

[18] J. B. Walters, A. M. Cruz-Uribe, H. R. Marschall, Sulfur loss from subducted altered oceanic crust and implications for mantle oxidation. Geochem. Perspect. Lett. 13, 36–41 (2020).

[19] Y. Zhang, E. Gazel, G. A. Gaetani, F. Klein, Serpentinite-derived slab fluids control the oxidation state of the subarc mantle. Sci. Adv. 7, eabj2515 (2021). 34826248 10.1126/sciadv.abj2515 PMC8626075

[20] B. Debret, D. A. Sverjensky, Highly oxidising fluids generated during serpentinite breakdown in subduction zones. Sci. Rep. 7, 10351 (2017). 28871200 10.1038/s41598-017-09626-y PMC5583334

[21] J.-L. Li, E. M. Schwarzenbach, T. John, J. J. Ague, F. Huang, J. Gao, R. Klemd, M. J. Whitehouse, X.-S. Wang, Uncovering and quantifying the subduction zone sulfur cycle from the slab perspective. Nat. Commun. 11, 514 (2020). 31980597 10.1038/s41467-019-14110-4 PMC6981181

[22] J. A. Padrón-Navarta, V. López Sánchez-Vizcaíno, M. D. Menzel, M. T. Gómez-Pugnaire, C. J. Garrido, Mantle wedge oxidation from deserpentinization modulated by sediment-derived fluids. Nat. Geosci. 16, 268–275 (2023).

[23] F. Piccoli, J. Hermann, T. Pettke, J. A. D. Connolly, E. D. Kempf, J. F. Vieira Duarte, Subducting serpentinites release reduced, not oxidized, aqueous fluids. Sci. Rep. 9, 19573 (2019). 31862932 10.1038/s41598-019-55944-8 PMC6925189

[24] J. J. Ague, S. Tassara, M. E. Holycross, J.-L. Li, E. Cottrell, E. M. Schwarzenbach, C. Fassoulas, T. John, Slab-derived devolatilization fluids oxidized by subducted metasedimentary rocks. Nat. Geosci. 15, 320–326 (2022).

[25] A. Maffeis, M. L. Frezzotti, J. A. D. Connolly, D. Castelli, S. Ferrando, Sulfur disproportionation in deep COHS slab fluids drives mantle wedge oxidation. Sci. Adv. 10, eadj2770 (2024). 38507499 10.1126/sciadv.adj2770 PMC10954224

[26] H. Moreira, C. Storey, E. Bruand, J. Darling, M. Fowler, M. Cotte, E. E. Villalobos-Portillo, F. Parat, L. Seixas, P. Philippot, Sub-arc mantle fugacity shifted by sediment recycling across the Great Oxidation Event. Nat. Geosci. 16, 922–927 (2023).

[27] F. Hu, H. Jiang, B. Wan, M. N. Ducea, L. Gao, F.-Y. Wu, Latitude-dependent oxygen fugacity in arc magmas. Nat. Commun. 15, 6050 (2024). 39025886 10.1038/s41467-024-50337-6 PMC11258285

[28] T. Plank, C. E. Manning, Subducting carbon. Nature 574, 343–352 (2019). 31619791 10.1038/s41586-019-1643-z

[29] C. J. Spencer, T. M. Gernon, N. S. Davies, W. J. McMahon, A. S. Merdith, From plant roots to mountain roots: Impact of land plants on arc magmatism. Geology 53, 679–683 (2025).

[30] M. W. Schmidt, O. Jagoutz, The global systematics of primitive arc melts. Geochem. Geophys. Geosyst. 18, 2817–2854 (2017).

[31] T. Plank, C. H. Langmuir, An evaluation of the global variations in the major element chemistry of arc basalts. Earth Planet. Sci. Lett. 90, 349–370 (1988).

[32] J. A. Pearce, D. W. Peate, Tectonic implications of the composition of volcanic arc magmas. Annu. Rev. Earth Planet. Sci. 23, 251–285 (1995).

[33] S. J. Turner, C. H. Langmuir, M. A. Dungan, S. Escrig, The importance of mantle wedge heterogeneity to subduction zone magmatism and the origin of EM1. Earth Planet. Sci. Lett. 472, 216–228 (2017).

[34] S. J. Turner, C. H. Langmuir, An evaluation of five models of arc volcanism. J. Petrol. 63, egac010 (2022).

[35] S. J. Turner, C. H. Langmuir, An alternative to the igneous crust fluid + sediment melt paradigm for arc lava geochemistry. Sci. Adv. 10, eadg6482 (2024). 38875329 10.1126/sciadv.adg6482 PMC11177931

[36] A. W. Hofmann, Mantle geochemistry: The message from oceanic volcanism. Nature 385, 219–229 (1997).

[37] R. Kessel, M. W. Schmidt, P. Ulmer, T. Pettke, Trace element signature of subduction-zone fluids, melts and supercritical liquids at 120-180 km depth. Nature 437, 724–727 (2005). 16193050 10.1038/nature03971

[38] R. K. Workman, S. R. Hart, Major and trace element composition of the depleted MORB mantle (DMM). Earth Planet. Sci. Lett. 231, 53–72 (2005).

[39] H. R. Marschall, “Boron isotopes in the ocean floor realm and the mantle,” in *Boron Isotopes: The Fifth Element*, H. Marschall, G. Foster, Eds. (Springer International Publishing, 2018), pp. 189–215.

[40] X.-Y. Qiao, J.-W. Xiong, Y.-X. Chen, J. C. M. De Hoog, J. Pearce, F. Huang, Z.-F. Zhao, K. Chen, Magnesium and boron isotope evidence for the generation of arc magma through serpentinite-mélange melting. Natl. Sci. Rev. 12, nwae363 (2025). 40395648 10.1093/nsr/nwae363 PMC12089755

[41] A. M. Rebaza, A. Mallik, E. H. G. Cooperdock, B. I. Holman, The fate of ultramafic-rich mélanges in cold to hot subduction zones: Implications for diapirism (or not) and chemical geodynamics. Earth Planet. Sci. Lett. 647, 119020 (2024).

[42] P. E. van Keken, B. R. Hacker, E. M. Syracuse, G. A. Abers, Subduction factory: 4. Depth-dependent flux of H_2_O from subducting slabs worldwide. J. Geophys. Res. Solid Earth 116, B01401 (2011).

[43] E. M. Syracuse, P. E. van Keken, G. A. Abers, The global range of subduction zone thermal models. Phys. Earth Planet. Inter. 183, 73–90 (2010).

[44] T. Plank, C. H. Langmuir, Tracing trace-elements from sediment input to volcanic output at subduction zones. Nature 362, 739–743 (1993).

[45] T. Plank, “4.17 - The chemical composition of subducting sediments,” in *Treatise on Geochemistry (Second Edition)*, H. D. Holland, K. K. Turekian, Eds. (Elsevier, 2014), pp. 607–629.

[46] F. Hauff, K. Hoernle, A. Schmidt, Sr-Nd-Pb composition of Mesozoic Pacific oceanic crust (site 1149 and 801, ODP Leg 185): Implications for alteration of ocean crust and the input into the Izu-Bonin-Mariana subduction system. Geochem. Geophys. Geosyst. 4, doi.org/10.1029/2002GC000421 (2003).

[47] H. Staudigel, G. R. Davies, S. R. Hart, K. M. Marchant, B. M. Smith, Large scale isotopic Sr, Nd and O isotopic anatomy of altered oceanic crust: DSDP/ODP sites 417/418. Earth Planet. Sci. Lett. 130, 169–185 (1995).

[48] J. Hermann, D. Rubatto, Accessory phase control on the trace element signature of sediment melts in subduction zones. Chem. Geol. 265, 512–526 (2009).

[49] S. Skora, J. Blundy, High-pressure hydrous phase relations of radiolarian clay and implications for the involvement of subducted sediment in arc magmatism. J. Petrol. 51, 2211–2243 (2010).

[50] J. M. Eiler, Oxygen isotope variations of basaltic lavas and upper mantle rocks. Rev. Mineral. Geochem. 43, 319–364 (2001).

[51] J. Hermann, C. J. Spandler, Sediment melts at sub-arc depths: An experimental study. J. Petrol. 49, 717–740 (2008).

[52] F. Sorbadere, P. Schiano, N. Métrich, E. Garaebiti, Insights into the origin of primitive silica-undersaturated arc magmas of Aoba volcano (Vanuatu arc). Contrib. Mineral. Petrol. 162, 995–1009 (2011).

[53] L. B. Cooper, D. M. Ruscitto, T. Plank, P. J. Wallace, E. M. Syracuse, C. E. Manning, Global variations in H_2_O/Ce: 1. Slab surface temperatures beneath volcanic arcs. Geochem. Geophys. Geosyst. 13, Q03024 (2012).

[54] K. J. Walowski, P. J. Wallace, M. A. Clynne, D. J. Rasmussen, D. Weis, Slab melting and magma formation beneath the southern Cascade arc. Earth Planet. Sci. Lett. 446, 100–112 (2016).

[55] T. Plank, C. H. Langmuir, The chemical composition of subducting sediment and its consequences for the crust and mantle. Chem. Geol. 145, 325–394 (1998).

[56] S. Song, S. Ye, M. B. Allen, Y. Niu, W. Sun, L. Zhang, Oxidation of arcs and mantle wedges by reduction of manganese in pelagic sediments during seafloor subduction. Am. Mineral. 107, 1850–1857 (2022).

[57] R. Arevalo Jr., W. F. McDonough, M. Luong, The K/U ratio of the silicate Earth: Insights into mantle composition, structure and thermal evolution. Earth Planet. Sci. Lett. 278, 361–369 (2009).

[58] J. D. Vervoort, T. Plank, J. Prytulak, The Hf–Nd isotopic composition of marine sediments. Geochim. Cosmochim. Acta 75, 5903–5926 (2011).

[59] Y. Wang, S. F. Foley, S. Buhre, J. Soldner, Y. Xu, Origin of potassic postcollisional volcanic rocks in young, shallow, blueschist-rich lithosphere. Sci. Adv. 7, eabc0291 (2021). 34261644 10.1126/sciadv.abc0291 PMC8279503

[60] S. Tommasini, R. Avanzinelli, S. Conticelli, The Th/La and Sm/La conundrum of the Tethyan realm lamproites. Earth Planet. Sci. Lett. 301, 469–478 (2011).

[61] S. M. Straub, A. Gómez-Tuena, P. Vannucchi, Subduction erosion and arc volcanism. Nat. Rev. Earth Environ. 1, 574–589 (2020).

[62] C.-T. Lee, W. P. Leeman, D. Canil, Z.-X. A. Li, Similar V/Sc systematics in MORB and Arc basalts: Implications for the oxygen fugacities of their mantle source regions. J. Petrol. 46, 2313–2336 (2005).

[63] C.-T. Liu, C.-Y. Ye, J. ZhangZhou, Modelling redox state via V-Sc-Ti-Yb partitioning in mantle derived melts. Geochem. Perspect. Lett. 33, 56–62 (2025).

[64] V. J. M. Salters, A. Stracke, Composition of the depleted mantle. Geochem. Geophys. Geosyst. 5, 1–27 (2004).

[65] M. Holycross, E. Cottrell, Experimental quantification of vanadium partitioning between eclogitic minerals (garnet, clinopyroxene, rutile) and silicate melt as a function of temperature and oxygen fugacity. Contrib. Mineral. Petrol. 177, 21 (2022).

[66] M. Gaborieau, M. Laubier, M. Pompilio, N. Bolfan-Casanova, Determination of the oxidation state of primary melts using two proxies. Chem. Geol. 638, 121701 (2023).

[67] K. A. Kelley, E. Cottrell, The influence of magmatic differentiation on the oxidation state of Fe in a basaltic arc magma. Earth Planet. Sci. Lett. 329-330, 109–121 (2012).

[68] C. E. Bucholz, G. A. Gaetani, M. D. Behn, N. Shimizu, Post-entrapment modification of volatiles and oxygen fugacity in olivine-hosted melt inclusions. Earth Planet. Sci. Lett. 374, 145–155 (2013).

[69] J. Humphreys, M. Brounce, K. Walowski, Diffusive equilibration of H_2_O and oxygen fugacity in natural olivine-hosted melt inclusions. Earth Planet. Sci. Lett. 584, 117409 (2022).

[70] M. J. Muth, P. J. Wallace, Sulfur recycling in subduction zones and the oxygen fugacity of mafic arc magmas. Earth Planet. Sci. Lett. 599, 117836 (2022).

[71] J. Blundy, E. Melekhova, L. Ziberna, M. Humphreys, V. Cerantola, R. A. Brooker, C. A. McCammon, M. Pichavant, P. Ulmer, Effect of redox on Fe–Mg–Mn exchange between olivine and melt and an oxybarometer for basalts. Contrib. Mineral. Petrol. 175, 103 (2020).

[72] P. Tollan, J. Hermann, Arc magmas oxidized by water dissociation and hydrogen incorporation in orthopyroxene. Nat. Geosci. 12, 667–671 (2019). 31372181 10.1038/s41561-019-0411-x PMC6675610

[73] P. J. Wallace, T. Plank, R. J. Bodnar, G. A. Gaetani, T. Shea, Olivine-hosted melt inclusions: A microscopic perspective on a complex magmatic world. Annu. Rev. Earth Planet. Sci. 49, 465–494 (2021).

[74] P. D. Clift, A revised budget for Cenozoic sedimentary carbon subduction. Rev. Geophys. 55, 97–125 (2017).

[75] M. S. Duncan, R. Dasgupta, Rise of Earth’s atmospheric oxygen controlled by efficient subduction of organic carbon. Nat. Geosci. 10, 387–392 (2017).

[76] K. A. Evans, A. G. Tomkins, The relationship between subduction zone redox budget and arc magma fertility. Earth Planet. Sci. Lett. 308, 401–409 (2011).

[77] R. Tao, Y. Fei, Recycled calcium carbonate is an efficient oxidation agent under deep upper mantle conditions. Commun. Earth Environ. 2, 45 (2021).

[78] M. Gao, Y. Wang, S. F. Foley, Y.-G. Xu, Variable mantle redox states driven by deeply subducted carbon. Sci. Adv. 11, eadu4985 (2025). 40397749 10.1126/sciadv.adu4985 PMC12094243

[79] Z. Zajacz, A. Tsay, An accurate model to predict sulfur concentration at anhydrite saturation in silicate melts. Geochim. Cosmochim. Acta 261, 288–304 (2019).

[80] Z. Xu, Y. Li, The sulfur concentration at anhydrite saturation in silicate melts: Implications for sulfur cycle and oxidation state in subduction zones. Geochim. Cosmochim. Acta 306, 98–123 (2021).

[81] H. Li, L. Zhang, X. Bao, J. L. Wykes, X. Liu, High sulfur solubility in subducted sediment melt under both reduced and oxidized conditions: With implications for S recycling in subduction zone settings. Geochim. Cosmochim. Acta 304, 305–326 (2021).

[82] C.-T. A. Lee, P. Luffi, E. J. Chin, R. Bouchet, R. Dasgupta, D. M. Morton, V. Le Roux, Q.-z. Yin, D. Jin, Copper systematics in arc magmas and implications for crust-mantle differentiation. Science 336, 64–68 (2012). 22491850 10.1126/science.1217313

[83] S. D’Hondt, F. Inagaki, C. A. Zarikian, L. J. Abrams, N. Dubois, T. Engelhardt, H. Evans, T. Ferdelman, B. Gribsholt, R. N. Harris, B. W. Hoppie, J.-H. Hyun, J. Kallmeyer, J. Kim, J. E. Lynch, C. C. McKinley, S. Mitsunobu, Y. Morono, R. W. Murray, R. Pockalny, J. Sauvage, T. Shimono, F. Shiraishi, D. C. Smith, C. E. Smith-Duque, A. J. Spivack, B. O. Steinsbu, Y. Suzuki, M. Szpak, L. Toffin, G. Uramoto, Y. T. Yamaguchi, G.-l. Zhang, X.-H. Zhang, W. Ziebis, Presence of oxygen and aerobic communities from sea floor to basement in deep-sea sediments. Nat. Geosci. 8, 299–304 (2015).

[84] J. Alt, J. Burdett, “Sulfur in Pacific deep-sea sediments (Leg 129) and implications for cycling of sediment in subduction zones,” in *Proceedings of the Ocean Drilling Program*. (Ocean Drilling Program, 1992), vol. 129, pp. 283–294.

[85] V. Galy, C. France-Lanord, O. Beyssac, P. Faure, H. Kudrass, F. Palhol, Efficient organic carbon burial in the Bengal fan sustained by the Himalayan erosional system. Nature 450, 407–410 (2007). 18004382 10.1038/nature06273

[86] C. Tiraboschi, C. McCammon, A. Rohrbach, S. Klemme, J. Berndt, C. Sanchez-Valle, Preferential mobilisation of oxidised iron by slab-derived hydrous silicate melts. Geochem. Perspect. Lett. 24, 43–47 (2023).

[87] K. A. Evans, Redox decoupling and redox budgets: Conceptual tools for the study of earth systems. Geology 34, 489–492 (2006).

[88] J.-W. Park, I. H. Campbell, M. Chiaradia, H. Hao, C.-T. Lee, Crustal magmatic controls on the formation of porphyry copper deposits. Nat. Rev. Earth Environ. 2, 542–557 (2021).

[89] Z. Hou, Z. Yang, X. Qu, X. Meng, Z. Li, G. Beaudoin, Z. Rui, Y. Gao, K. Zaw, The Miocene Gangdese porphyry copper belt generated during post-collisional extension in the Tibetan Orogen. Ore Geol. Rev. 36, 25–51 (2009).

[90] C. G. Soder, J. Dunga, R. L. Romer, Continental subduction controls regional magma heterogeneity and distribution of porphyry deposits in post-collisional settings. Geochim. Cosmochim. Acta 375, 217–228 (2024).

[91] Z. Yang, X. Sun, M. Chiaradia, Y. Lu, R. Yin, Z. Hou, H. Li, Y. Zhou, Oxidized sediment recycling as a driver for postsubduction porphyry copper formation. Sci. Adv. 11, eadx4474 (2025). 40601748 10.1126/sciadv.adx4474 PMC12219508

[92] H. Li, Z. Yang, Y. Lu, Z. Hou, Redox state of subducted sediments controls porphyry copper mineralization along the Tethyan belt. Nat. Commun. 16, 6456 (2025). 40651970 10.1038/s41467-025-61668-3 PMC12255737

[93] D. Canil, S. A. Fellows, Sulphide–sulphate stability and melting in subducted sediment and its role in arc mantle redox and chalcophile cycling in space and time. Earth Planet. Sci. Lett. 470, 73–86 (2017).

[94] Y.-C. Zheng, S.-A. Liu, C.-D. Wu, W. L. Griffin, Z.-Q. Li, B. Xu, Z.-M. Yang, Z.-Q. Hou, S. Y. O’Reilly, Cu isotopes reveal initial Cu enrichment in sources of giant porphyry deposits in a collisional setting. Geology 47, 135–138 (2019).

[95] J. Chang, A. Audétat, T. Pettke, The gold content of mafic to felsic potassic magmas. Nat. Commun. 15, 6988 (2024). 39143075 10.1038/s41467-024-51405-7 PMC11324659

[96] X. Liu, L. Li, T. Xu, X. Xiong, J. Wang, Z. Wang, H. S. C. O’Neill, Gold solubility enhanced by H_2_O in sulfur-bearing magma: Implications for gold partitioning and mineralization. Geochim. Cosmochim. Acta 393, 170–181 (2025).

[97] M. Gao, H. Xu, J. Zhang, S. F. Foley, Experimental interaction of granitic melt and peridotite at 1.5 GPa: Implications for the origin of post-collisional K-rich magmatism in continental subduction zones. Lithos 350-351, 105241 (2019).

[98] P. Condamine, E. Médard, Experimental melting of phlogopite-bearing mantle at 1 GPa: Implications for potassic magmatism. Earth Planet. Sci. Lett. 397, 80–92 (2014).

[99] C. Shu, S. F. Foley, I. S. Ezad, N. R. Daczko, S. S. Shcheka, Experimental melting of phlogopite websterite in the upper mantle between 1.5 and 4.5 GPa. J. Petrol. 65, egae030 (2024).

[100] A. Gale, C. A. Dalton, C. H. Langmuir, Y. Su, J.-G. Schilling, The mean composition of ocean ridge basalts. Geochem. Geophys. Geosyst. 14, 489–518 (2013).

[101] D. M. Ruscitto, P. J. Wallace, L. B. Cooper, T. Plank, Global variations in H_2_O/Ce: 2. Relationships to arc magma geochemistry and volatile fluxes. Geochem. Geophys. Geosyst. 13, Q03025 (2012).

[102] U. Mann, M. W. Schmidt, Melting of pelitic sediments at subarc depths: 1. Flux vs. fluid-absent melting and a parameterization of melt productivity. Chem. Geol. 404, 150–167 (2015).

[103] Y. Wang, D. Prelević, S. Buhre, S. F. Foley, Constraints on the sources of post-collisional K-rich magmatism: The roles of continental clastic sediments and terrigenous blueschists. Chem. Geol. 455, 192–207 (2017).

[104] D. McKenzie, R. K. O’Nions, Partial melt distributions from inversion of rare earth element concentrations. J. Petrol. 32, 1021–1091 (1991).

[105] S. Erdmann, M. Pichavant, F. Gaillard, Mineral-melt vanadium oxybarometry for primitive arc magmas: Effect of hydrous melt composition on *f*O_2_ estimates. Contrib. Mineral. Petrol. 179, 39 (2024).

[106] T. Plank, K. A. Kelley, M. M. Zimmer, E. H. Hauri, P. J. Wallace, Why do mafic arc magmas contain ~4 wt% water on average? Earth Planet. Sci. Lett. 364, 168–179 (2013).

[107] B. O. Mysen, D. Virgo, F. A. Seifert, The structure of silicate melts: Implications for chemical and physical properties of natural magma. Rev. Geophys. 20, 353–383 (1982).

[108] K. D. Putirka, M. Perfit, F. J. Ryerson, M. G. Jackson, Ambient and excess mantle temperatures, olivine thermometry, and active vs. passive upwelling. Chem. Geol. 241, 177–206 (2007).

[109] Z. Sun, X. Xiong, J. Wang, X. Liu, L. Li, M. Ruan, L. Zhang, E. Takahashi, Sulfur abundance and heterogeneity in the MORB mantle estimated by copper partitioning and sulfur solubility modelling. Earth Planet. Sci. Lett. 538, 116169 (2020).

[110] Z.-X. Anser Li, C.-T. A. Lee, The constancy of upper mantle fO_2_ through time inferred from V/Sc ratios in basalts. Earth Planet. Sci. Lett. 228, 483–493 (2004).

[111] D. A. Singer, V. I. Berger, B. C. Moring, “Porphyry copper deposits of the world: Database and grade and tonnage models, 2008” (Open-File Report, 2008-1155, US Geological Survey, 2008).

[112] W. Frisch, “Morphology across convergent plate boundaries,” in *Encyclopedia of Marine Geosciences*, J. Harff, M. Meschede, S. Petersen, J. Thiede, Eds. (Springer Netherlands, 2013), pp. 1–7.

[113] S. H. Kirby, S. Stein, E. A. Okal, D. C. Rubie, Metastable mantle phase transformations and deep earthquakes in subducting oceanic lithosphere. Rev. Geophys. 34, 261–306 (1996).

[114] H. S. C. O’Neill, The smoothness and shapes of chondrite-normalized rare earth element patterns in basalts. J. Petrol. 57, 1463–1508 (2016).

[115] S. Lallemand, D. Arcay, Subduction initiation from the earliest stages to self-sustained subduction: Insights from the analysis of 70 Cenozoic sites. Earth Sci. Rev. 221, 103779 (2021).

[116] M. Willbold, A. Stracke, Formation of enriched mantle components by recycling of upper and lower continental crust. Chem. Geol. 276, 188–197 (2010).

[117] D. Ben Othman, W. M. White, J. Patchett, The geochemistry of marine sediments, island arc magma genesis, and crust-mantle recycling. Earth Planet. Sci. Lett. 94, 1–21 (1989).

[118] W. M. White, B. Dupré, P. Vidal, Isotope and trace element geochemistry of sediments from the Barbados Ridge-Demerara Plain region, Atlantic Ocean. Geochim. Cosmochim. Acta 49, 1875–1886 (1985).

[119] H. R. Marschall, V. D. Wanless, N. Shimizu, P. A. E. Pogge von Strandmann, T. Elliott, B. D. Monteleone, The boron and lithium isotopic composition of mid-ocean ridge basalts and the mantle. Geochim. Cosmochim. Acta 207, 102–138 (2017).

