# Supplementary material for: Progressive sub-arc mantle oxidation modulated by sediment melt
Source: Sci Adv. 2026 Apr 3;12(14):eaeb9023. doi: 10.1126/sciadv.aeb9023 (PMC13240789; doi:10.1126/sciadv.aeb9023)
Supplement: Supplementary file 1 — Supplementary Text Figs. S1 to S16 Table S1 Legends for data S1 to S6 References [file sciadv.aeb9023_sm.pdf]

Science Advances
AAAS

Supplementary Materials for

Progressive sub-arc mantle oxidation modulated by sediment melt

Mingdi Gao *et al.*

Corresponding author: Yu Wang, wangyu@gig.ac.cn

Sci. Adv. **12**, eaeb9023 (2026)
DOI: 10.1126/sciadv.aeb9023

The PDF file includes:

Supplementary Text
Figs. S1 to S16
Table S1
Legends for data S1 to S6
References

Other Supplementary Material for this manuscript includes the following:

Data S1 to S6

Trace element distribution during partial melting of sediment melt modified mantle

In Fig. 2, the shaded area is the range of melt compositions that derived from sources of depleted MORB mantle (DMM) hybridized by 1-10 wt% sediment melt. The DMM and sediment melt trace element compositions are from Workman and Hart *(38)* and Hermann and Rubatto (*48*), respectively, in which the sediment melt trace element compositions were re-calibrated based on GLOSS composition as the starting composition. Melting of the hybridized mantle was modeled based on a simple batch melting model, where element partition coefficients of the bulk residue and melt are from Workman and Hart *(38)*.

In the modelling results, the lower limit in Fig. 2 is from a source of DMM+1 wt% low-temperature, low-degree melt, corresponding to the scenario occurred in cold intra-oceanic arcs. Both the low incompatible element contents in low temperature melt, and low fractions of melt incorporated into the mantle result in minimal trace element enrichment. On the other hand, the upper limit is from a source of DMM+10 wt% high temperature, high-degree melt, corresponding to the scenario occurred in warm to hot continental arcs. Detailed calculation process and results are listed in data S6.

Quantitative estimation of sediment melt influx into the sub-arc mantle

The flux of sediment melt into the sub-arc mantle for per meter arc length was calculated according to:

$$M_{sed} = D_{sed} \times v_{slab} \times F_{sed} \quad (7)$$

where D_{sed} and v_{slab} are the subducted sediment thickness and slab subduction velocity, respectively. Sediment melting degree (F_{sed}) was calculated based on the parameterization of Mann and Schmidt (*102*) under water-saturated condition:

$$F_{sed} = -90.1 + 13.05 ln(c_{H_2O}) + 0.1405T - 3.62 \times 10^{-6}T^2 - 5.01P \quad (8)$$

where T and P represent the slab surface temperature and pressure at sub-arc depths. The water content in the sediment (c_{H_2O}) was assumed to be 7 wt% (*102*). The values of T, P, along with D_{sed} and v_{slab} in each arc are from Syracuse *et al. (43)*. The calculation results show that the sediment melt influx lie between 2-20×10^6 g/year/m, with continental arcs

generally have elevated sediment melt influx. Detailed calculation process and results are listed in data S1.

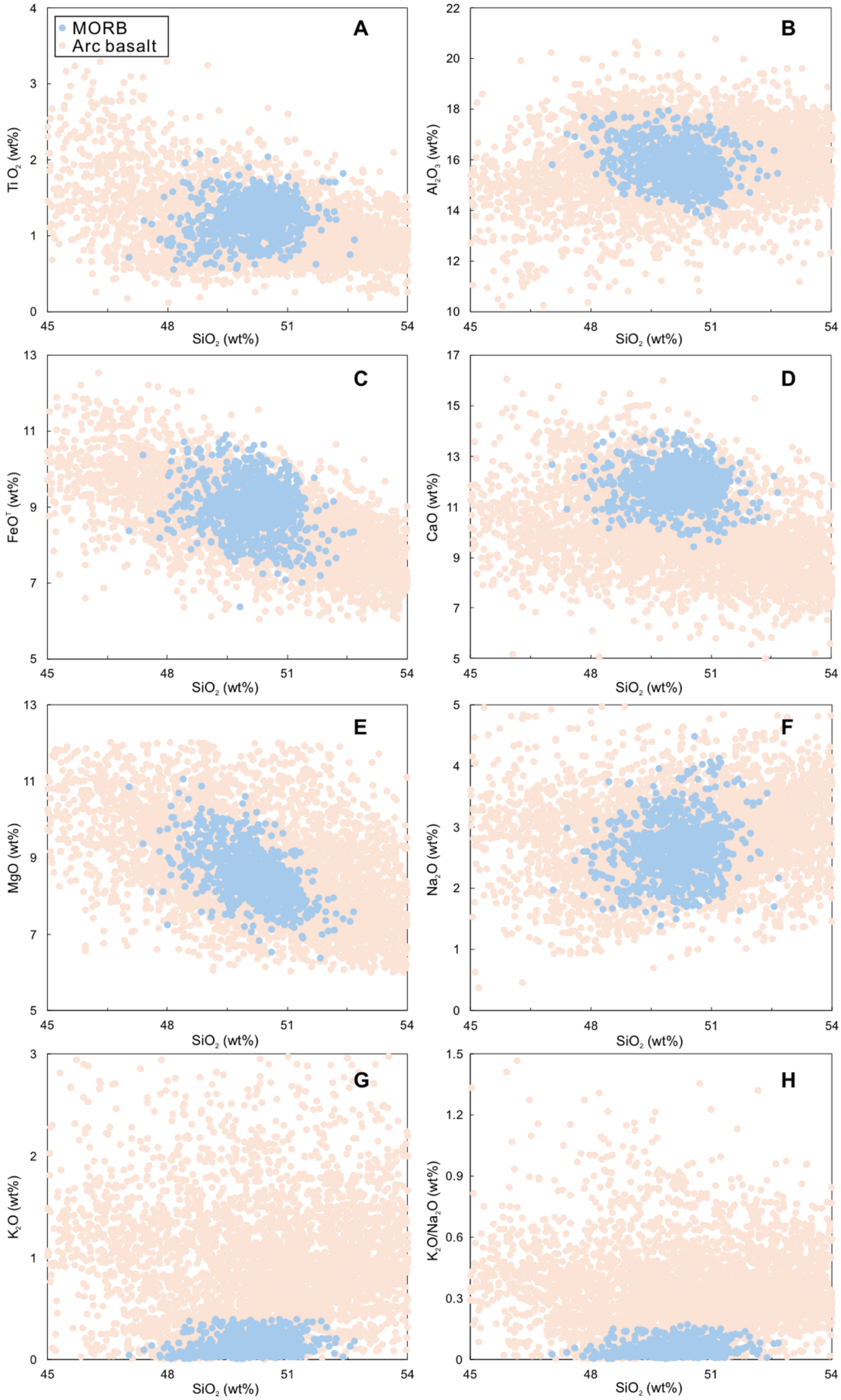

Fig. S1. Major element composition comparison between primitive MORBs and arc basalts. Most components are consistent between MORBs and arc basalts **(A-F)**, except for K_2O **(G)** and K_2O/Na_2O ratio **(H)**.

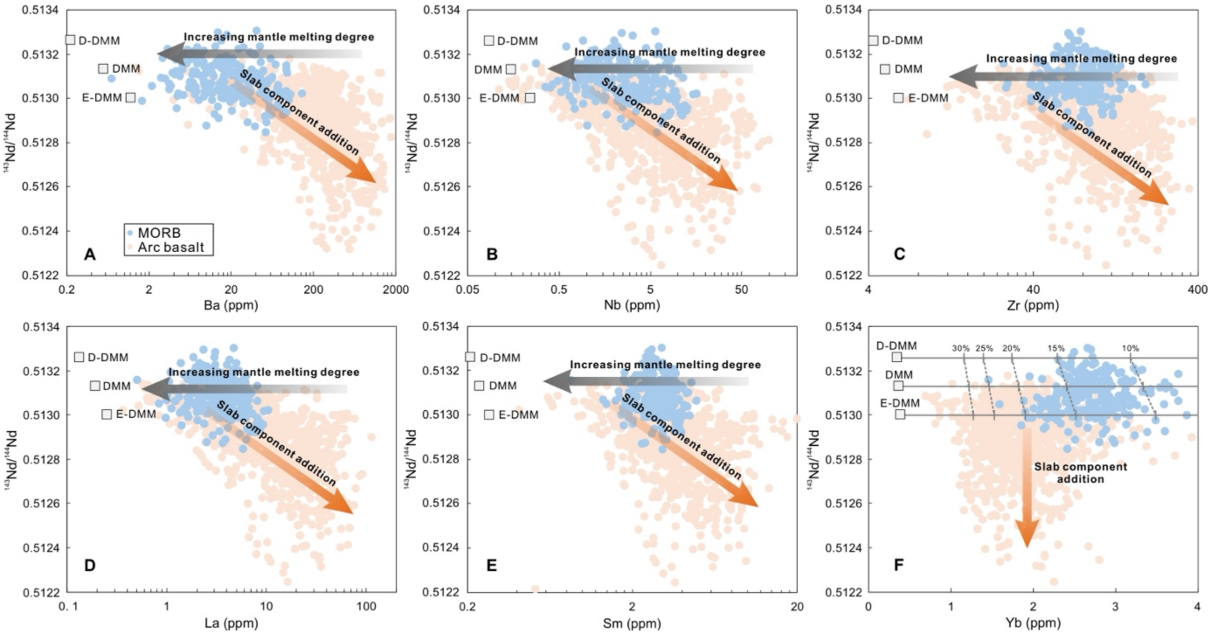

Fig. S2. Variations of LILE (Ba), HFSE (Nb and Zr) and REE (La, Sm, Yb) with ^{143}Nd/^{144}Nd in primitive MORBs and arc basalts. Concentrations of LILEs **(A)**, HFSEs **(B to C)**, and light to middle REEs **(D to E)** in arc basalts are positively correlated with ^{143}Nd/^{144}Nd, indicating a contribution from slab components to their mantle source. In contrast, heavy REE (Yb) content **(F)** does not increase with decreasing ^{143}Nd/^{144}Nd, confirming that Yb is primarily derived from the ambient mantle. Compositions of depleted MORB mantle (DMM), enriched DMM (E-DMM), and depleted DMM (D-DMM) are from Workman and Hart (*38*).

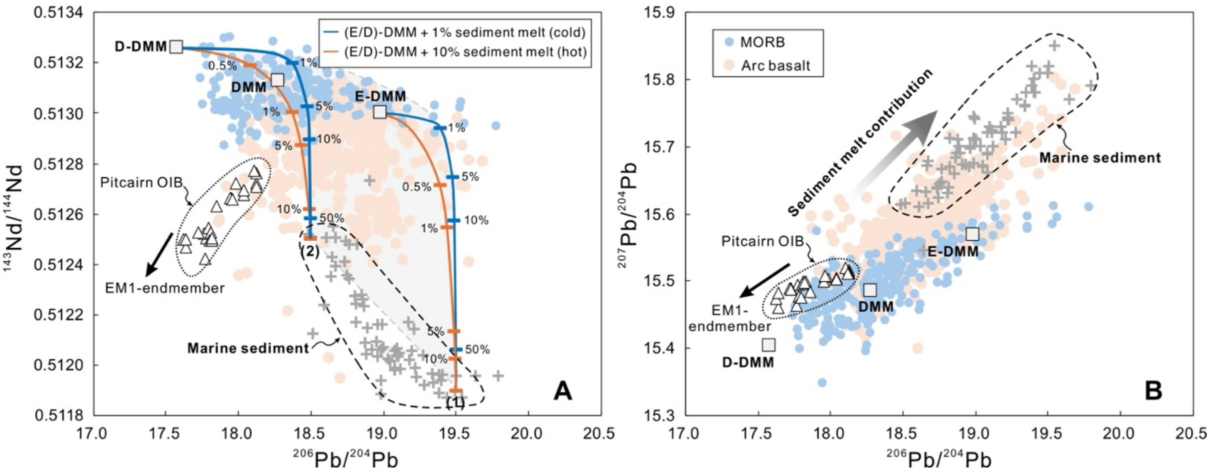

Fig. S3. Nd and Pb isotope variations in primitive MORBs and arc basalts. (A) Partial melting of various DMM (ranging from D-DMM to E-DMM) hybridized with sediment melt of varying composition and proportion (grey field) can reproduce the ^{143}Nd/^{144}Nd *vs.* ^{206}Pb/^{204}Pb variations observed in most global arc basalts. In contrast, the ^{206}Pb/^{204}Pb value in the EM-1 mantle endmember is too low to account for the values in most arc basalts. **(B)** The ^{206}Pb/^{204}Pb *vs.* ^{207}Pb/^{204}Pb variations in arc basalts range from MORB-like signatures to elevated values, whereas the characteristically low Pb isotope ratios of the EM-1 mantle are not widely observed in arc basalts. Modelling utilized sediment melt endmembers with the following two isotopic compositions: (1) ^{206}Pb/^{204}Pb = 19.5, ^{143}Nd/^{144}Nd = 0.5119 and (2) ^{206}Pb/^{204}Pb = 18.5, ^{143}Nd/^{144}Nd = 0.5125. Number near the modelling curve indicate the melting degree of the sediment melt hybridized mantle. Data of Pitcairn OIBs (sourced from the EM-1 mantle) are from Willbold and Stracke (*116*), marine sediments are from Ben Othman *et al.* (*117*) and White *et al.* (*118*).

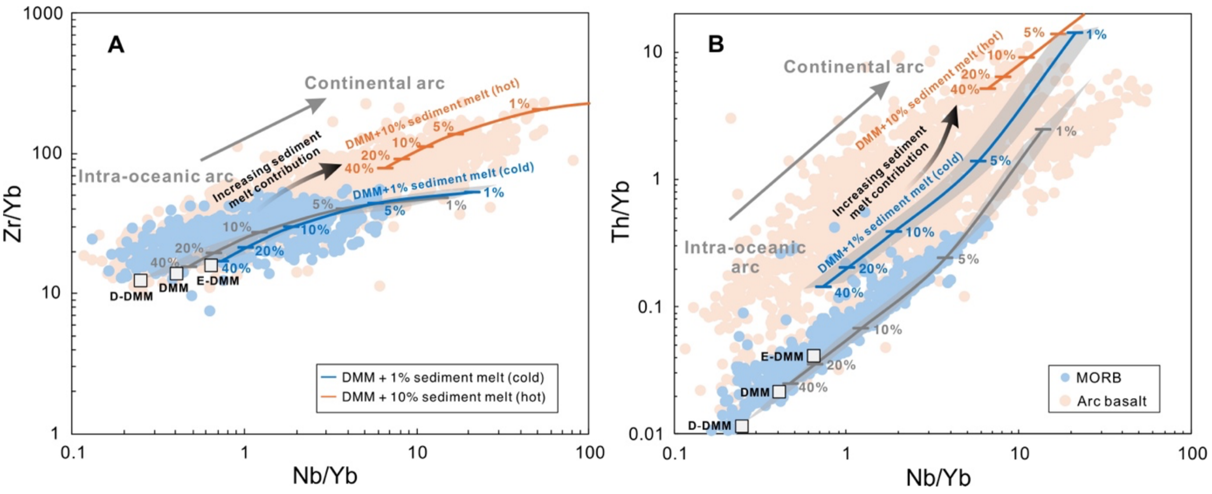

Fig. S4. The "Pearce diagram" (*32*) for primitive MORBs and arc basalts. Arc basalts define an array similar to MORBs in Nb/Yb *vs.* Zr/Yb space **(A)** but exhibit notably elevated Th/Yb ratios, particularly in continental arc magmas **(B)**. Given the immobility of Yb during slab dehydration and melting, the elevated Th/Yb ratios in arc magmas indicate a substantial Th contribution from sediment melt. However, the similar arrays in **(A)** do not imply that Nb and Zr are immobile. Partial melting modeling of a depleted MORB mantle (DMM) plus 1-10% sediment melt reproduces the Nb/Yb *vs.* Zr/Yb variations observed in arc basalts. The results indicate that sediment melts carry Nb and Zr at similar levels, producing correlated increases in both Nb/Yb and Zr/Yb and preserving an array comparable to that of MORBs. Compositions of the "cold" and "hot" sediment melts are from the sediment melting experiments of Hermann and Rubatto *(48)* at relatively low and high temperatures, respectively. The shaded grey areas represent the uncertainty propagated from different mantle source compositions (from D-DMM to E-DMM) (see data S6 for calculation detail). Number near the modelling curve indicate the melting degree of the sediment melt hybridized mantle.

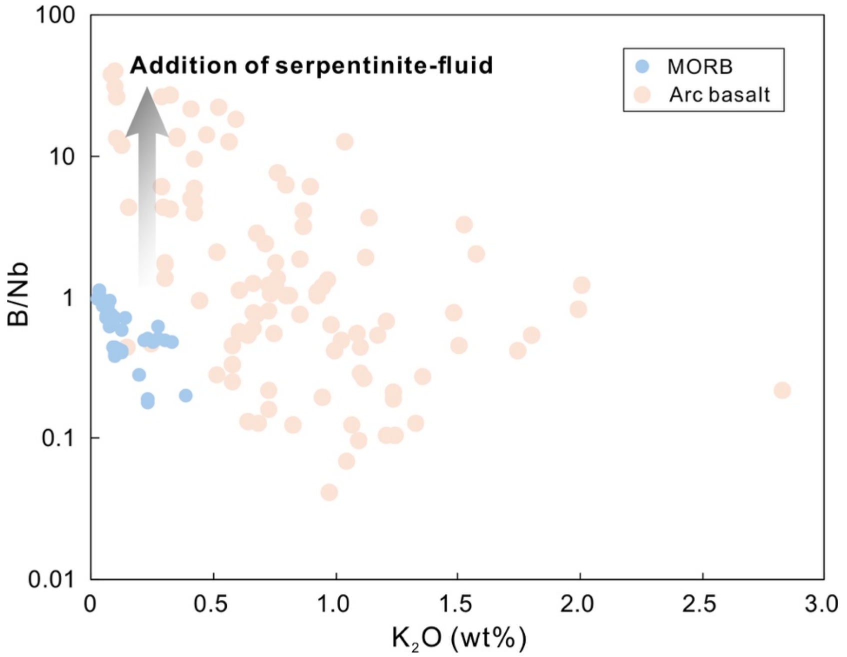

Fig. S5. Variations of B/Nb with K_2O in primitive MORBs and arc basalts. The elevated B/Nb ratios of low-K_2O arc basalts signal a substantial contribution from serpentinite-derived fluids. MORB B/Nb values are from Marschall et al. (*119*)

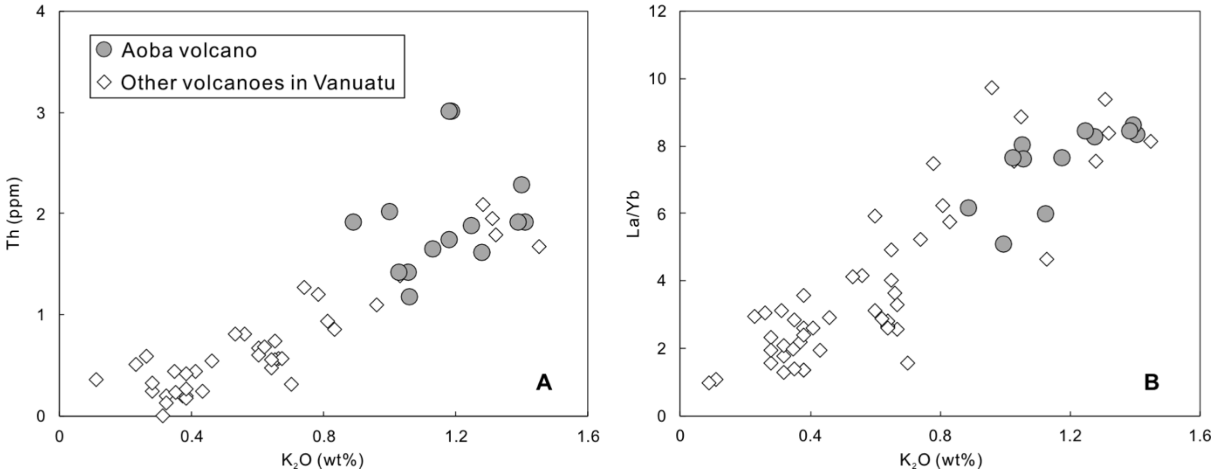

Fig. S6. Comparison of the compositions of Aoba basalts in North Vanuatu and basalts from other localities of the Vanuatu arc. The Aoba magmas are characterized by high K_2O, high Th **(A)** and elevated La/Yb ratios **(B)**, indicating substantial sediment melt contribution in their source.

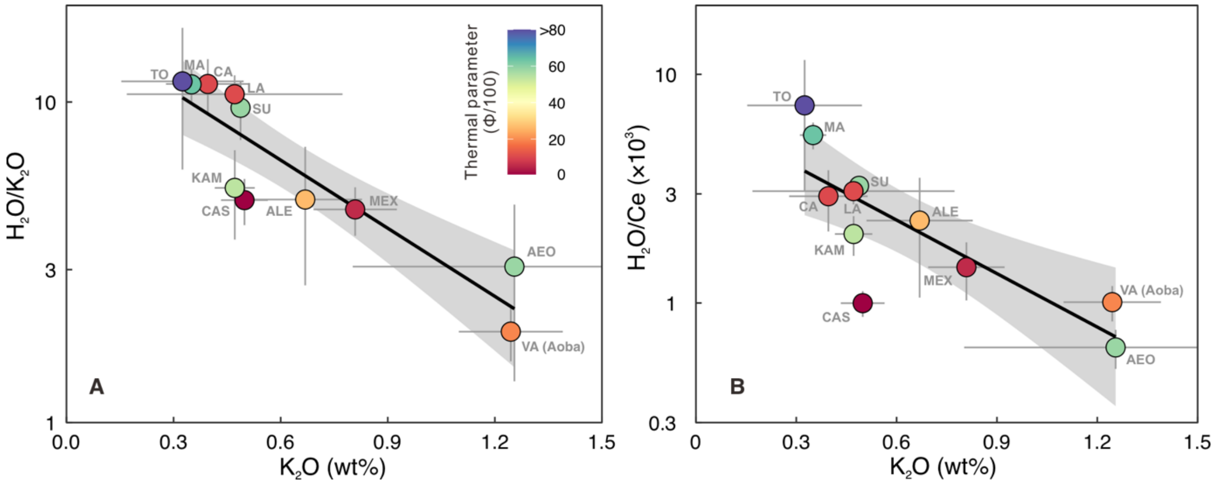

Fig. S7. Variations of melt inclusion H_2O/K_2O (A) and H_2O/Ce (B) ratios with inclusion K_2O contents.

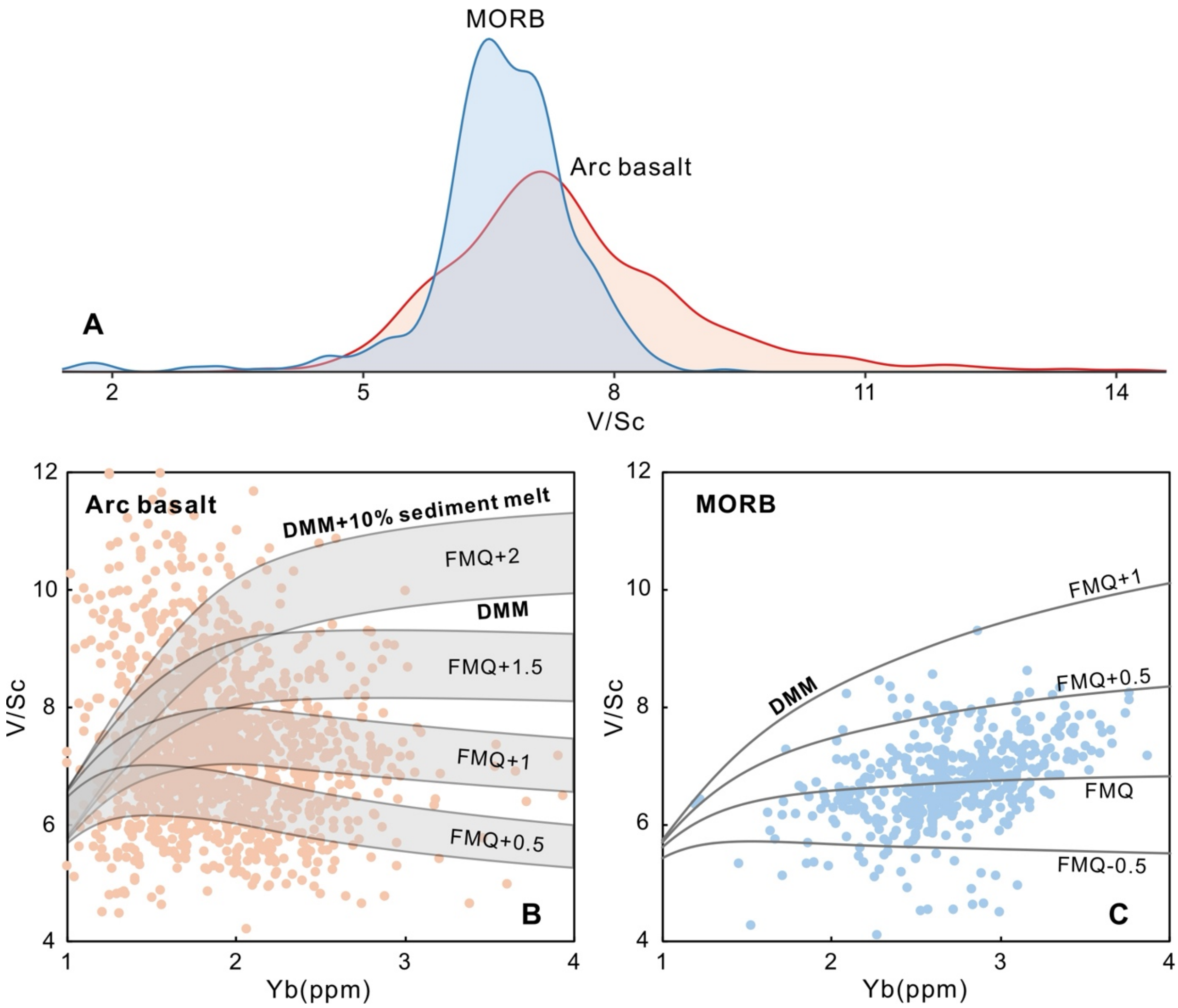

Fig. S8. Comparison of V/Sc ratios in primitive MORBs and arc basalts. The frequency histogram shows that arc basalts have comparable but more scattered V/Sc ratios compared to MORBs **(A)**. Due to the relatively lower melting temperature, arc basalts show higher fO_2 values at given V/Sc ratios **(B)** compared in MORBs **(C)**. Yb in **(B-C)** are used as an index for mantle melting degree, where the Yb content decreases with increasing melting degree. Detailed calculation process for the variation of V/Sc with Yb at a certain fO_2 are provided in data S4.

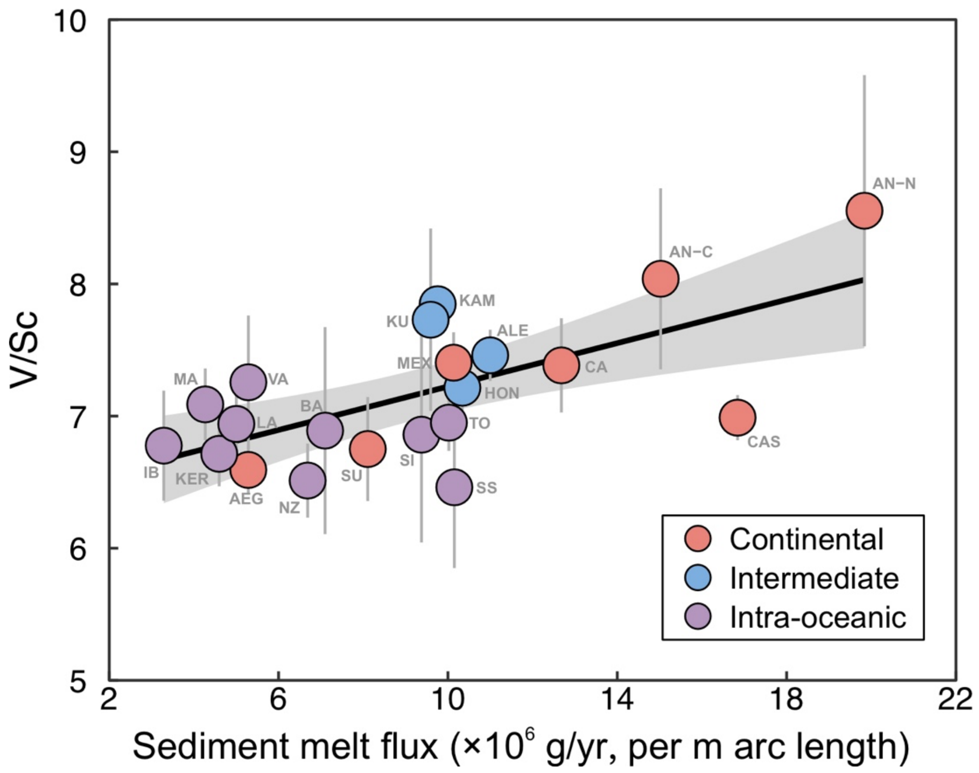

Fig. S9. Variations of V/Sc in arc basalts with the sediment melt flux into mantle wedge. The positive correlation may indicate increasing fO_2 with increasing sediment melt contribution in the source. Detailed calculation process for sediment melt flux is provided in the Supplementary text and data S1.

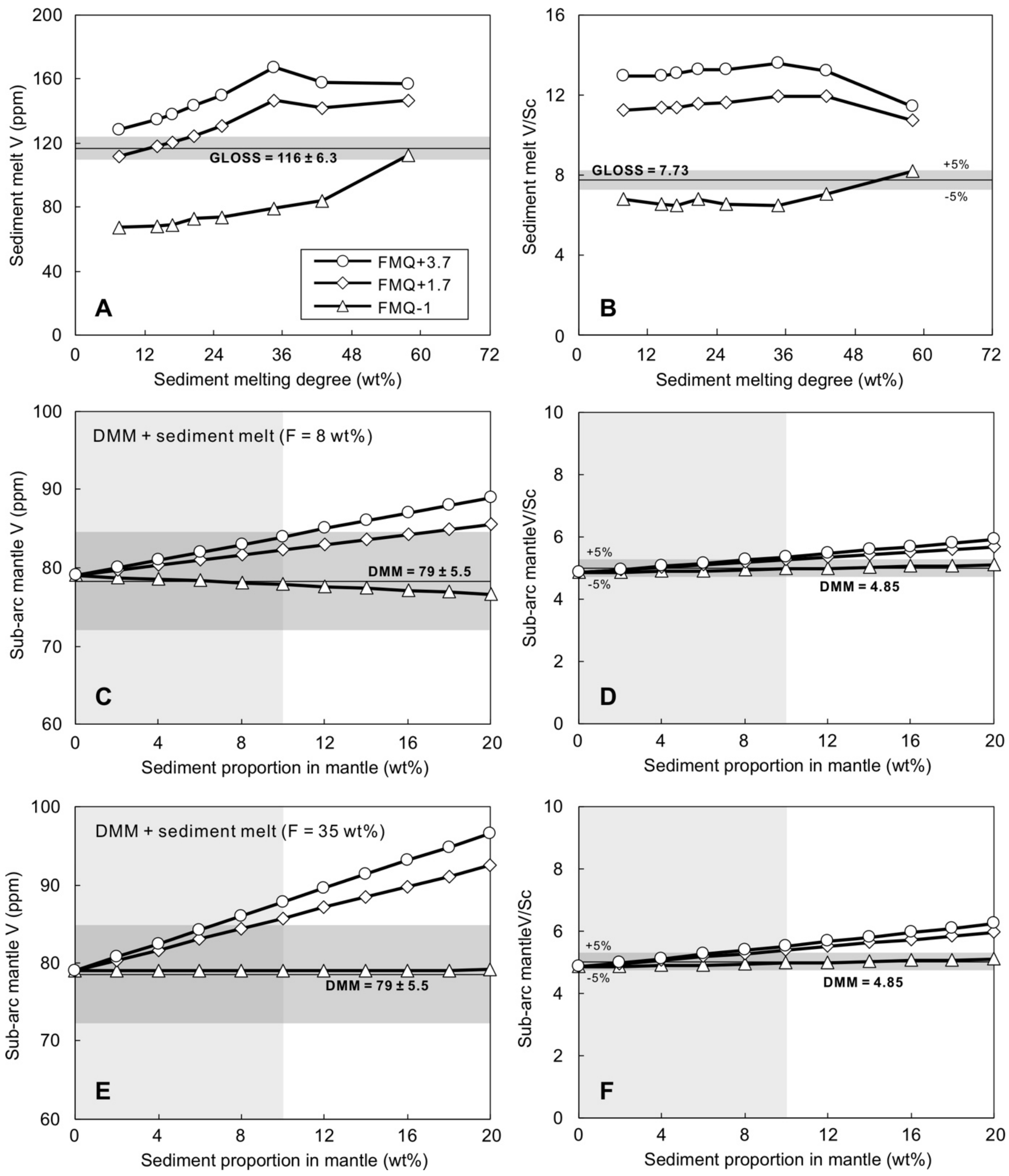

Fig. S10. Melt V content and V/Sc ratio variations during sediment partial melting (A-B), and sub-arc mantle V and V/Sc variations caused by sediment melt contribution at various *f*O_2 conditions (C-F). Detailed calculation process is provided in data S5.

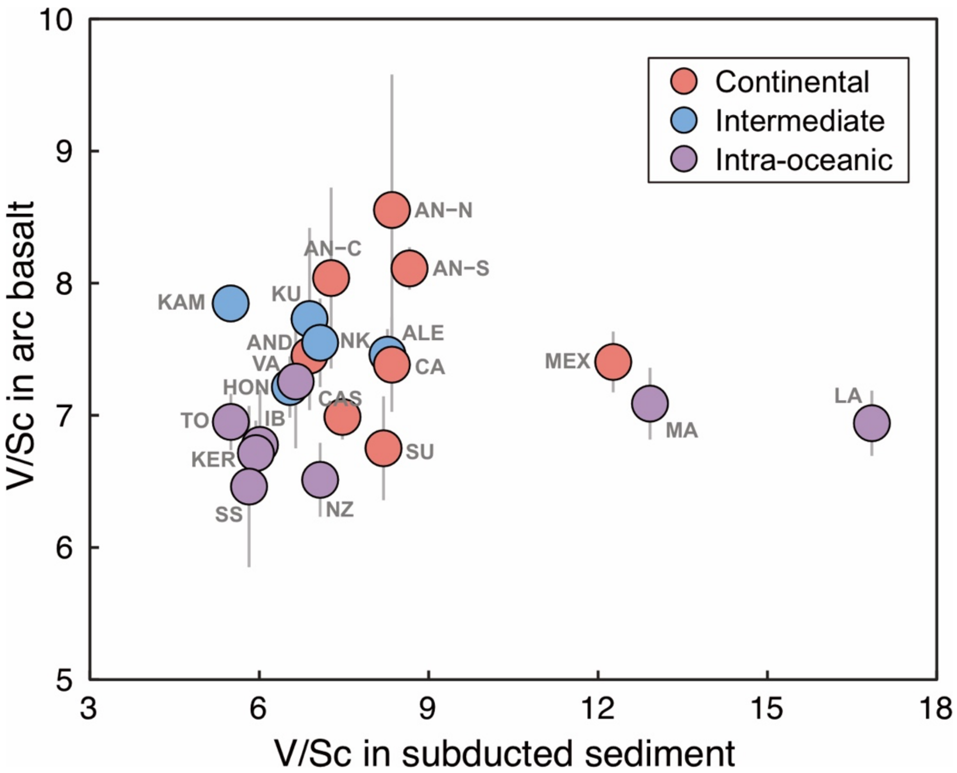

Fig. S11. The relationship between V/Sc in the subducted sediment (input) and in arc basalts (output). The lack of correlation indicates that the subducted sediments do not contribute notably excess V and Sc into the sub-arc mantle. V/Sc ratio of the subducted sediment in each arc segment is from Plank (*45*).

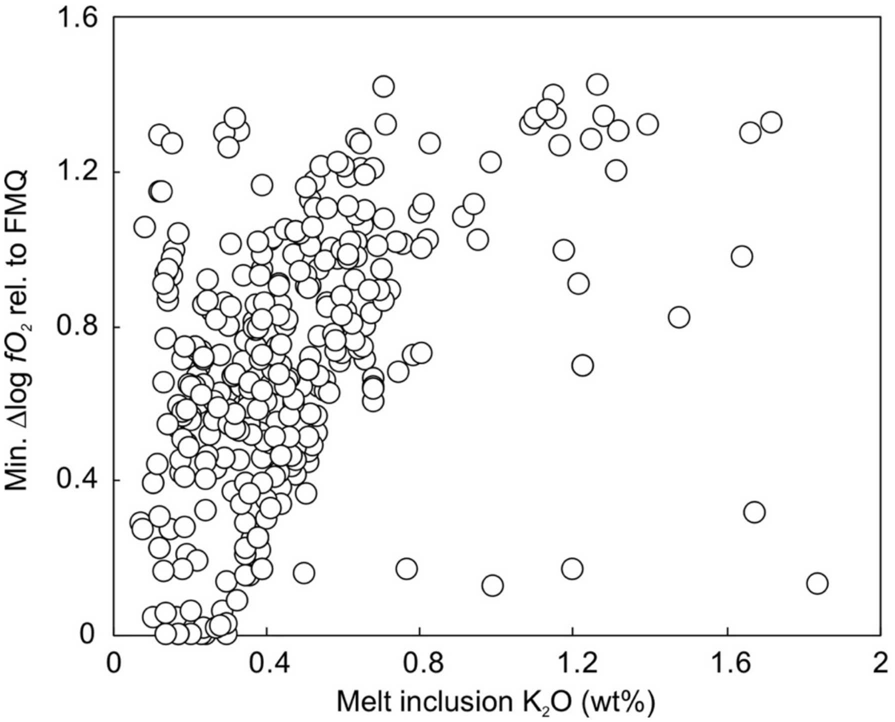

Fig. S12. Positive correlation between the melt inclusion K_2O content and the estimated minimum fO_2 by inclusion sulfur content. Original data and the calculation method of minimum fO_2 are from Muth and Wallace (*70*).

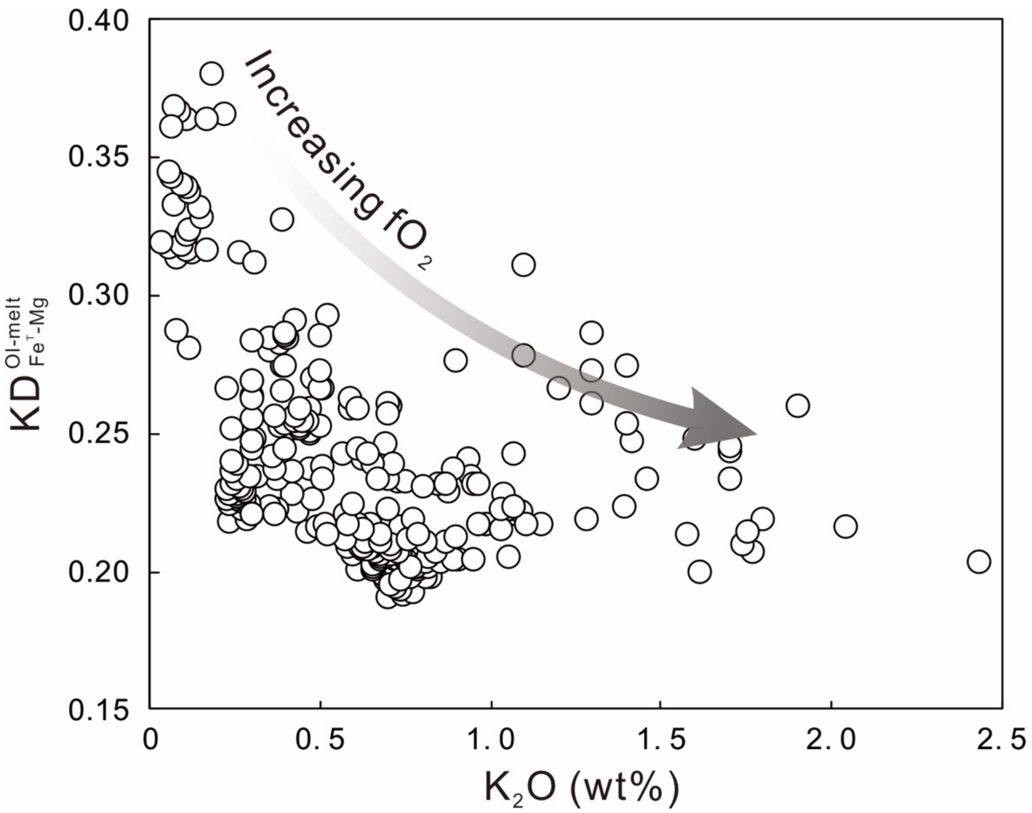

Fig. S13. Host olivine-melt inclusion Fe^T(total iron)-Mg exchange coefficient ($KD_{Fe^T-Mg}^{Ol-melt}$) variation with melt inclusion K_2O content. Fe^{2+} is compatible, while Fe^{3+} is highly compatible in olivine, and thus the $KD_{Fe^T-Mg}^{Ol-melt}$ value would decrease with increasing fO_2 condition (*71*). The negative correlation between $KD_{Fe^T-Mg}^{Ol-melt}$ and inclusion K_2O content suggests the highly oxidized feature of the high K_2O arc magma. $KD_{Fe^T-Mg}^{Ol-melt} = (Fe_{Ol}/Fe_{melt}) \times (Mg_{melt}/Mg_{Ol})$.

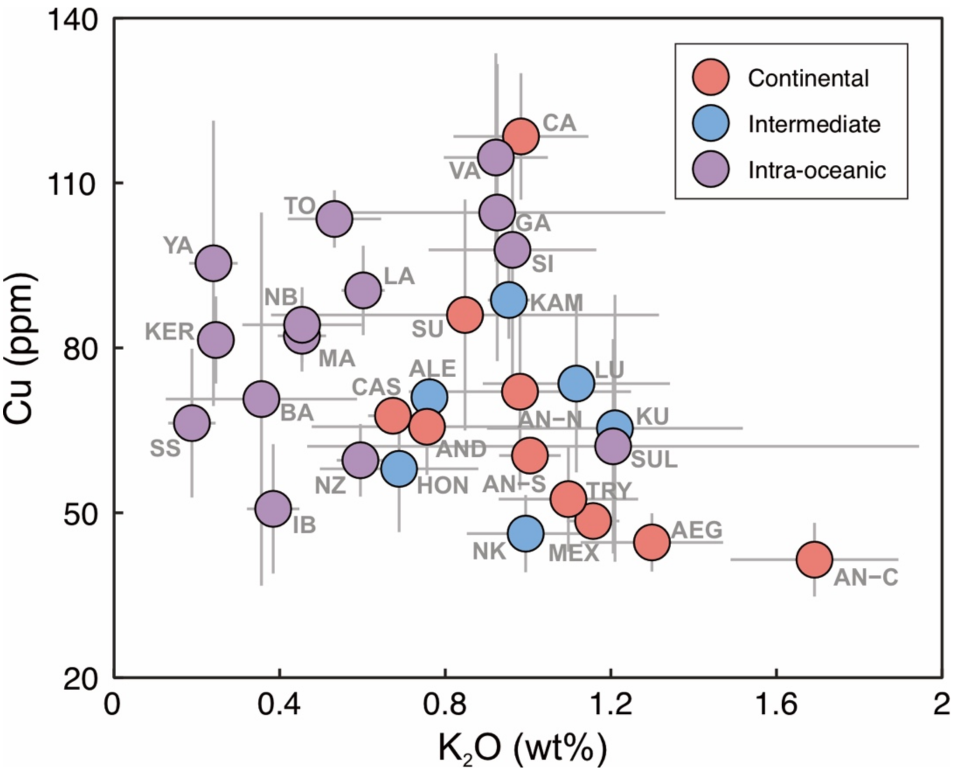

Fig. S14. The lack of correlation between arc basalt Cu content and K_2O content, showing that source enrichment is not a requirement for the formation of porphyry Cu-Au deposits.

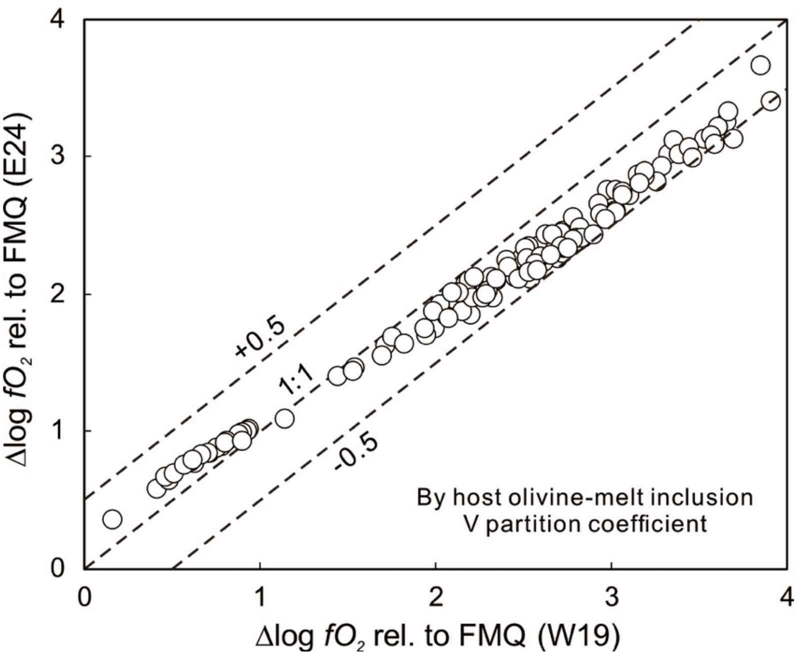

267 **Fig. S15. Comparison of calculated fO$_2$ values by using different empirical equations.** The
268 empirical equations are from W19-Wang *et al.* (*3*) and E24-Erdmann *et al.* (*105*).

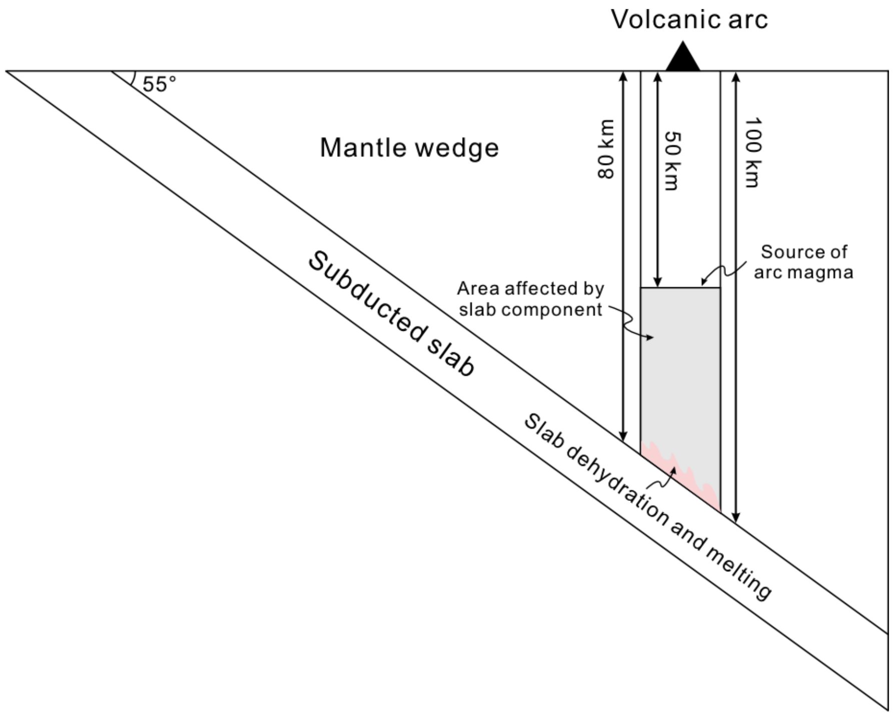

Fig. S16. Schematic diagram of possible area affected by sediment melt in a 2-dimensional space (the grey area). A subduction angle of 55º, sub-arc depth range of 80-100 km, and volcanic arc source depth of 50 km are used for estimation.

Table S1. Parameters and their ranges used for the redox budget calculation

Parameter	Intra-oceanic arc	Continental arc	References
Subducted slab velocity (v_{slab})	0.04-0.07 m/year	0.04-0.07 m/year	(*43*)
Subducted sediment thickness (D_{sed})	200-600 m	300-1000 m	(*43*)
Sediment melting degree (F_{sed})	10-30%	20-40%	(*102*)
S^{6+} in sediment melt	0.01 wt%	0.04 wt%	(*79, 80*)
Fe^{3+} in sediment melt	0.04-0.16 wt%	0.69-1.11 wt%	(*86*)
mass of mantle wedge affected by slab sediment melt (per m arc length) (M)	1.77×10^{12}kg/m	1.77×10^{12}kg/m	fig. S16

Other Supplementary Materials for this manuscript include the following
(Captions for Data S1 to S6**):**

Data S1. Compiled global arc basalt compositions. Raw data were obtained from the GOEROC database (https://georoc.mpch-mainz.gwdg.de/georoc/) on 5 November 2023, using the following parameters: geological setting = convergent margins. This database is distributed under the CC BY SA license. Detailed criteria for data filtration are described in the Materials and Methods section.

Data S2. Compiled global mid-ocean ridge basalt compositions. Raw data were obtained from Gale, A., Dalton, C. A., Langmuir, C. H., Su, Y., & Schilling, J-G. Geochemistry, Geophysics, Geosystems. Published by John Wiley and Sons, Inc. *(100)*. © 2013. American Geophysical Union. All Rights Reserved. Reproduced with permission. License No. 6195070067988. Criteria for data filtration are described in the Materials and Methods section.

Data S3. Compiled olivine-hosted melt inclusion data, with calculated fO_2 values by different methods.

Data S4. Melt V/Sc content calculation during sub-arc mantle and MORB mantle melting at various fO_2 conditions.

Data S5. Melt V/Sc content calculation during sediment melting at various fO_2 conditions.

Data S6. Calculation of melt trace element compositions and Nd-Pb isotopes during melting of sediment melt hybridized mantle.

REFERENCES

1. E. Cottrell, S. K. Birner, M. Brounce, F. A. Davis, L. E. Waters, K. A. Kelley, “Oxygen fugacity across tectonic settings,” in *Magma Redox Geochemistry*, R. Moretti, D. R. Neuville, Eds. (Wiley, 2021), pp. 33–61.

2. K. A. Kelley, E. Cottrell, Water and the oxidation state of subduction zone magmas. *Science* **325**, 605–607 (2009).

3. J. Wang, X. Xiong, E. Takahashi, L. Zhang, L. Li, X. Liu, Oxidation state of arc mantle revealed by partitioning of V, Sc, and Ti between mantle minerals and basaltic melts. *J. Geophys. Res. Solid Earth* **124**, 4617–4638 (2019).

4. K. A. Evans, The redox budget of subduction zones. *Earth Sci. Rev.* **113**, 11–32 (2012).

5. J. P. Richards, The oxidation state, and sulfur and Cu contents of arc magmas: Implications for metallogeny. *Lithos* **233**, 27–45 (2015).

6. W. Sun, R.-f. Huang, H. Li, Y.-b. Hu, C.-c. Zhang, S.-j. Sun, L.-p. Zhang, X. Ding, C.-y. Li, R. E. Zartman, M.-x. Ling, Porphyry deposits and oxidized magmas. *Ore Geol. Rev.* **65**, 97–131 (2015).

7. C.-T. A. Lee, M. Tang, How to make porphyry copper deposits. *Earth Planet. Sci. Lett.* **529**, 115868 (2020).

8. M. Tang, M. Erdman, G. Eldridge, C.-T. A. Lee, The redox “filter” beneath magmatic orogens and the formation of continental crust. *Sci. Adv.* **4**, eaar4444 (2018).

9. I. J. Parkinson, R. J. Arculus, The redox state of subduction zones: Insights from arc-peridotites. *Chem. Geol.* **160**, 409–423 (1999).

10. S.-Y. Zhao, A. Y. Yang, C. H. Langmuir, T.-P. Zhao, Oxidized primary arc magmas: Constraints from Cu/Zr systematics in global arc volcanics. *Sci. Adv.* **8**, eabk0718 (2022).

11. T. Elliott, “Tracers of the slab,” in *Inside the Subduction Factory*, J. Eiler, Ed. (American Geophysical Union, 2003), pp. 23–45.

12. H. Li, J. Hermann, L. Zhang, Melting of subducted slab dictates trace element recycling in global arcs. *Sci. Adv.* **8**, eabh2166 (2022).

13. S. J. Turner, C. H. Langmuir, Sediment and ocean crust both melt at subduction zones. *Earth Planet. Sci. Lett.* **584**, 117424 (2022).

14. E. A. Codillo, V. Le Roux, H. R. Marschall, Arc-like magmas generated by mélange-peridotite interaction in the mantle wedge. *Nat. Commun.* **9**, 2864 (2018).

15. S. G. Nielsen, H. R. Marschall, Geochemical evidence for mélange melting in global arcs. *Sci. Adv.* **3**, e1602402 (2017).

16. A. M. Cruz-Uribe, H. R. Marschall, G. A. Gaetani, V. Le Roux, Generation of alkaline magmas in subduction zones by partial melting of mélange diapirs—An experimental study. *Geology* **46**, 343–346 (2018).

17. L. M. Saper, M. Brounce, D. Woelki, R. Cao, G. Bromiley, Variable oxidizing capacity of slab-derived fluids: Insights from Fe and S speciation in glasses from the Troodos Ophiolite. *Earth Planet. Sci. Lett.* **627**, 118560 (2024).

18. J. B. Walters, A. M. Cruz-Uribe, H. R. Marschall, Sulfur loss from subducted altered oceanic crust and implications for mantle oxidation. *Geochem. Perspect. Lett.* **13**, 36–41 (2020).

19. Y. Zhang, E. Gazel, G. A. Gaetani, F. Klein, Serpentinite-derived slab fluids control the oxidation state of the subarc mantle. *Sci. Adv.* **7**, eabj2515 (2021).

20. B. Debret, D. A. Sverjensky, Highly oxidising fluids generated during serpentinite breakdown in subduction zones. *Sci. Rep.* **7**, 10351 (2017).

21. J.-L. Li, E. M. Schwarzenbach, T. John, J. J. Ague, F. Huang, J. Gao, R. Klemd, M. J. Whitehouse, X.-S. Wang, Uncovering and quantifying the subduction zone sulfur cycle from the slab perspective. *Nat. Commun.* **11**, 514 (2020).

22. J. A. Padrón-Navarta, V. López Sánchez-Vizcaíno, M. D. Menzel, M. T. Gómez-Pugnaire, C. J. Garrido, Mantle wedge oxidation from deserpentinization modulated by sediment-derived fluids. *Nat. Geosci.* **16**, 268–275 (2023).

23. F. Piccoli, J. Hermann, T. Pettke, J. A. D. Connolly, E. D. Kempf, J. F. Vieira Duarte, Subducting serpentinites release reduced, not oxidized, aqueous fluids. *Sci. Rep.* **9**, 19573 (2019).

24. J. J. Ague, S. Tassara, M. E. Holycross, J.-L. Li, E. Cottrell, E. M. Schwarzenbach, C. Fassoulas, T. John, Slab-derived devolatilization fluids oxidized by subducted metasedimentary rocks. *Nat. Geosci.* **15**, 320–326 (2022).

25. A. Maffeis, M. L. Frezzotti, J. A. D. Connolly, D. Castelli, S. Ferrando, Sulfur disproportionation in deep COHS slab fluids drives mantle wedge oxidation. *Sci. Adv.* **10**, eadj2770 (2024).

26. H. Moreira, C. Storey, E. Bruand, J. Darling, M. Fowler, M. Cotte, E. E. Villalobos-Portillo, F. Parat, L. Seixas, P. Philippot, Sub-arc mantle fugacity shifted by sediment recycling across the Great Oxidation Event. *Nat. Geosci.* **16**, 922–927 (2023).

27. F. Hu, H. Jiang, B. Wan, M. N. Ducea, L. Gao, F.-Y. Wu, Latitude-dependent oxygen fugacity in arc magmas. *Nat. Commun.* **15**, 6050 (2024).

28. T. Plank, C. E. Manning, Subducting carbon. *Nature* **574**, 343–352 (2019).

29. C. J. Spencer, T. M. Gernon, N. S. Davies, W. J. McMahon, A. S. Merdith, From plant roots to mountain roots: Impact of land plants on arc magmatism. *Geology* **53**, 679–683 (2025).

30. M. W. Schmidt, O. Jagoutz, The global systematics of primitive arc melts. *Geochem. Geophys. Geosyst.* **18**, 2817–2854 (2017).

31. T. Plank, C. H. Langmuir, An evaluation of the global variations in the major element chemistry of arc basalts. *Earth Planet. Sci. Lett.* **90**, 349–370 (1988).

32. J. A. Pearce, D. W. Peate, Tectonic implications of the composition of volcanic arc magmas. *Annu. Rev. Earth Planet. Sci.* **23**, 251–285 (1995).

33. S. J. Turner, C. H. Langmuir, M. A. Dungan, S. Escrig, The importance of mantle wedge heterogeneity to subduction zone magmatism and the origin of EM1. *Earth Planet. Sci. Lett.* **472**, 216–228 (2017).

34. S. J. Turner, C. H. Langmuir, An evaluation of five models of arc volcanism. *J. Petrol.* **63**, egac010 (2022).

35. S. J. Turner, C. H. Langmuir, An alternative to the igneous crust fluid + sediment melt paradigm for arc lava geochemistry. *Sci. Adv.* **10**, eadg6482 (2024).

36. A. W. Hofmann, Mantle geochemistry: The message from oceanic volcanism. *Nature* **385**, 219–229 (1997).

37. R. Kessel, M. W. Schmidt, P. Ulmer, T. Pettke, Trace element signature of subduction-zone fluids, melts and supercritical liquids at 120-180 km depth. *Nature* **437**, 724–727 (2005).

38. R. K. Workman, S. R. Hart, Major and trace element composition of the depleted MORB mantle (DMM). *Earth Planet. Sci. Lett.* **231**, 53–72 (2005).

39. H. R. Marschall, "Boron isotopes in the ocean floor realm and the mantle," in *Boron Isotopes: The Fifth Element*, H. Marschall, G. Foster, Eds. (Springer International Publishing, 2018), pp. 189–215.

40. X.-Y. Qiao, J.-W. Xiong, Y.-X. Chen, J. C. M. De Hoog, J. Pearce, F. Huang, Z.-F. Zhao, K. Chen, Magnesium and boron isotope evidence for the generation of arc magma through serpentinite-mélange melting. *Natl. Sci. Rev.* **12**, nwae363 (2025).

41. A. M. Rebaza, A. Mallik, E. H. G. Cooperdock, B. I. Holman, The fate of ultramafic-rich mélanges in cold to hot subduction zones: Implications for diapirism (or not) and chemical geodynamics. *Earth Planet. Sci. Lett.* **647**, 119020 (2024).

42. P. E. van Keken, B. R. Hacker, E. M. Syracuse, G. A. Abers, Subduction factory: 4. Depth-dependent flux of H_2O from subducting slabs worldwide. *J. Geophys. Res. Solid Earth* **116**, B01401 (2011).

43. E. M. Syracuse, P. E. van Keken, G. A. Abers, The global range of subduction zone thermal models. *Phys. Earth Planet. Inter.* **183**, 73–90 (2010).

44. T. Plank, C. H. Langmuir, Tracing trace-elements from sediment input to volcanic output at subduction zones. *Nature* **362**, 739–743 (1993).

45. T. Plank, "4.17 - The chemical composition of subducting sediments," in *Treatise on Geochemistry (Second Edition)*, H. D. Holland, K. K. Turekian, Eds. (Elsevier, 2014), pp. 607–629.

46. F. Hauff, K. Hoernle, A. Schmidt, Sr-Nd-Pb composition of Mesozoic Pacific oceanic crust (site 1149 and 801, ODP Leg 185): Implications for alteration of ocean crust and the input into the Izu-Bonin-Mariana subduction system. *Geochem. Geophys. Geosyst.* **4**, doi.org/10.1029/2002GC000421 (2003).

47. H. Staudigel, G. R. Davies, S. R. Hart, K. M. Marchant, B. M. Smith, Large scale isotopic Sr, Nd and O isotopic anatomy of altered oceanic crust: DSDP/ODP sites 417/418. *Earth Planet. Sci. Lett.* **130**, 169–185 (1995).

48. J. Hermann, D. Rubatto, Accessory phase control on the trace element signature of sediment melts in subduction zones. *Chem. Geol.* **265**, 512–526 (2009).

49. S. Skora, J. Blundy, High-pressure hydrous phase relations of radiolarian clay and implications for the involvement of subducted sediment in arc magmatism. *J. Petrol.* **51**, 2211–2243 (2010).

50. J. M. Eiler, Oxygen isotope variations of basaltic lavas and upper mantle rocks. *Rev. Mineral. Geochem.* **43**, 319–364 (2001).

51. J. Hermann, C. J. Spandler, Sediment melts at sub-arc depths: An experimental study. *J. Petrol.* **49**, 717–740 (2008).

52. F. Sorbadere, P. Schiano, N. Métrich, E. Garaebiti, Insights into the origin of primitive silica-undersaturated arc magmas of Aoba volcano (Vanuatu arc). *Contrib. Mineral. Petrol.* **162**, 995–1009 (2011).

53. L. B. Cooper, D. M. Ruscitto, T. Plank, P. J. Wallace, E. M. Syracuse, C. E. Manning, Global variations in H_2O/Ce: 1. Slab surface temperatures beneath volcanic arcs. *Geochem. Geophys. Geosyst.* **13**, Q03024 (2012).

54. K. J. Walowski, P. J. Wallace, M. A. Clynne, D. J. Rasmussen, D. Weis, Slab melting and magma formation beneath the southern Cascade arc. *Earth Planet. Sci. Lett.* **446**, 100–112 (2016).

55. T. Plank, C. H. Langmuir, The chemical composition of subducting sediment and its consequences for the crust and mantle. *Chem. Geol.* **145**, 325–394 (1998).

56. S. Song, S. Ye, M. B. Allen, Y. Niu, W. Sun, L. Zhang, Oxidation of arcs and mantle wedges by reduction of manganese in pelagic sediments during seafloor subduction. *Am. Mineral.* **107**, 1850–1857 (2022).

57. R. Arevalo Jr, W. F. McDonough, M. Luong, The K/U ratio of the silicate Earth: Insights into mantle composition, structure and thermal evolution. *Earth Planet. Sci. Lett.* **278**, 361–369 (2009).

58. J. D. Vervoort, T. Plank, J. Prytulak, The Hf–Nd isotopic composition of marine sediments. *Geochim. Cosmochim. Acta* **75**, 5903–5926 (2011).

59. Y. Wang, S. F. Foley, S. Buhre, J. Soldner, Y. Xu, Origin of potassic postcollisional volcanic rocks in young, shallow, blueschist-rich lithosphere. *Sci. Adv.* **7**, eabc0291 (2021).

60. S. Tommasini, R. Avanzinelli, S. Conticelli, The Th/La and Sm/La conundrum of the Tethyan realm lamproites. *Earth Planet. Sci. Lett.* **301**, 469–478 (2011).

61. S. M. Straub, A. Gómez-Tuena, P. Vannucchi, Subduction erosion and arc volcanism. *Nat. Rev. Earth Environ.* **1**, 574–589 (2020).

62. C.-T. Lee, W. P. Leeman, D. Canil, Z.-X. A. Li, Similar V/Sc systematics in MORB and Arc basalts: Implications for the oxygen fugacities of their mantle source regions. *J. Petrol.* **46**, 2313–2336 (2005).

63. C.-T. Liu, C.-Y. Ye, J. ZhangZhou, Modelling redox state via V-Sc-Ti-Yb partitioning in mantle derived melts. *Geochem. Perspect. Lett.* **33**, 56–62 (2025).

64. V. J. M. Salters, A. Stracke, Composition of the depleted mantle. *Geochem. Geophys. Geosyst.* **5**, 1–27 (2004).

65. M. Holycross, E. Cottrell, Experimental quantification of vanadium partitioning between eclogitic minerals (garnet, clinopyroxene, rutile) and silicate melt as a function of temperature and oxygen fugacity. *Contrib. Mineral. Petrol.* **177**, 21 (2022).

66. M. Gaborieau, M. Laubier, M. Pompilio, N. Bolfan-Casanova, Determination of the oxidation state of primary melts using two proxies. *Chem. Geol.* **638**, 121701 (2023).

67. K. A. Kelley, E. Cottrell, The influence of magmatic differentiation on the oxidation state of Fe in a basaltic arc magma. *Earth Planet. Sci. Lett.* **329-330**, 109–121 (2012).

68. C. E. Bucholz, G. A. Gaetani, M. D. Behn, N. Shimizu, Post-entrapment modification of volatiles and oxygen fugacity in olivine-hosted melt inclusions. *Earth Planet. Sci. Lett.* **374**, 145–155 (2013).

69. J. Humphreys, M. Brounce, K. Walowski, Diffusive equilibration of H_2O and oxygen fugacity in natural olivine-hosted melt inclusions. *Earth Planet. Sci. Lett.* **584**, 117409 (2022).

70. M. J. Muth, P. J. Wallace, Sulfur recycling in subduction zones and the oxygen fugacity of mafic arc magmas. *Earth Planet. Sci. Lett.* **599**, 117836 (2022).

71. J. Blundy, E. Melekhova, L. Ziberna, M. Humphreys, V. Cerantola, R. A. Brooker, C. A. McCammon, M. Pichavant, P. Ulmer, Effect of redox on Fe–Mg–Mn exchange between olivine and melt and an oxybarometer for basalts. *Contrib. Mineral. Petrol.* **175**, 103 (2020).

72. P. Tollan, J. Hermann, Arc magmas oxidized by water dissociation and hydrogen incorporation in orthopyroxene. *Nat. Geosci.* **12**, 667–671 (2019).

73. P. J. Wallace, T. Plank, R. J. Bodnar, G. A. Gaetani, T. Shea, Olivine-hosted melt inclusions: A microscopic perspective on a complex magmatic world. *Annu. Rev. Earth Planet. Sci.* **49**, 465–494 (2021).

74. P. D. Clift, A revised budget for Cenozoic sedimentary carbon subduction. *Rev. Geophys.* **55**, 97–125 (2017).

75. M. S. Duncan, R. Dasgupta, Rise of Earth's atmospheric oxygen controlled by efficient subduction of organic carbon. *Nat. Geosci.* **10**, 387–392 (2017).

76. K. A. Evans, A. G. Tomkins, The relationship between subduction zone redox budget and arc magma fertility. *Earth Planet. Sci. Lett.* **308**, 401–409 (2011).

77. R. Tao, Y. Fei, Recycled calcium carbonate is an efficient oxidation agent under deep upper mantle conditions. *Commun. Earth Environ.* **2**, 45 (2021).

78. M. Gao, Y. Wang, S. F. Foley, Y.-G. Xu, Variable mantle redox states driven by deeply subducted carbon. *Sci. Adv.* **11**, eadu4985 (2025).

79. Z. Zajacz, A. Tsay, An accurate model to predict sulfur concentration at anhydrite saturation in silicate melts. *Geochim. Cosmochim. Acta* **261**, 288–304 (2019).

80. Z. Xu, Y. Li, The sulfur concentration at anhydrite saturation in silicate melts: Implications for sulfur cycle and oxidation state in subduction zones. *Geochim. Cosmochim. Acta* **306**, 98–123 (2021).

81. H. Li, L. Zhang, X. Bao, J. L. Wykes, X. Liu, High sulfur solubility in subducted sediment melt under both reduced and oxidized conditions: With implications for S recycling in subduction zone settings. *Geochim. Cosmochim. Acta* **304**, 305–326 (2021).

82. C.-T. A. Lee, P. Luffi, E. J. Chin, R. Bouchet, R. Dasgupta, D. M. Morton, V. Le Roux, Q.-z. Yin, D. Jin, Copper systematics in arc magmas and implications for crust-mantle differentiation. *Science* **336**, 64–68 (2012).

83. S. D'Hondt, F. Inagaki, C. A. Zarikian, L. J. Abrams, N. Dubois, T. Engelhardt, H. Evans, T. Ferdelman, B. Gribsholt, R. N. Harris, B. W. Hoppie, J.-H. Hyun, J. Kallmeyer, J. Kim, J. E. Lynch, C. C. McKinley, S. Mitsunobu, Y. Morono, R. W. Murray, R. Pockalny, J. Sauvage, T. Shimono, F. Shiraishi, D. C. Smith, C. E. Smith-Duque, A. J. Spivack, B. O. Steinsbu, Y. Suzuki, M. Szpak, L. Toffin, G. Uramoto, Y. T. Yamaguchi, G.-l. Zhang, X.-H. Zhang, W. Ziebis, Presence of oxygen and aerobic communities from sea floor to basement in deep-sea sediments. *Nat. Geosci.* **8**, 299–304 (2015).

84. J. Alt, J. Burdett, "Sulfur in Pacific deep-sea sediments (Leg 129) and implications for cycling of sediment in subduction zones," in *Proceedings of the Ocean Drilling Program*. (Ocean Drilling Program, 1992), vol. 129, pp. 283–294.

85. V. Galy, C. France-Lanord, O. Beyssac, P. Faure, H. Kudrass, F. Palhol, Efficient organic carbon burial in the Bengal fan sustained by the Himalayan erosional system. *Nature* **450**, 407–410 (2007).

86. C. Tiraboschi, C. McCammon, A. Rohrbach, S. Klemme, J. Berndt, C. Sanchez-Valle, Preferential mobilisation of oxidised iron by slab-derived hydrous silicate melts. *Geochem. Perspect. Lett.* **24**, 43–47 (2023).

87. K. A. Evans, Redox decoupling and redox budgets: Conceptual tools for the study of earth systems. *Geology* **34**, 489–492 (2006).

88. J.-W. Park, I. H. Campbell, M. Chiaradia, H. Hao, C.-T. Lee, Crustal magmatic controls on the formation of porphyry copper deposits. *Nat. Rev. Earth Environ.* **2**, 542–557 (2021).

89. Z. Hou, Z. Yang, X. Qu, X. Meng, Z. Li, G. Beaudoin, Z. Rui, Y. Gao, K. Zaw, The Miocene Gangdese porphyry copper belt generated during post-collisional extension in the Tibetan Orogen. *Ore Geol. Rev.* **36**, 25–51 (2009).

90. C. G. Soder, J. Dunga, R. L. Romer, Continental subduction controls regional magma heterogeneity and distribution of porphyry deposits in post-collisional settings. *Geochim. Cosmochim. Acta* **375**, 217–228 (2024).

91. Z. Yang, X. Sun, M. Chiaradia, Y. Lu, R. Yin, Z. Hou, H. Li, Y. Zhou, Oxidized sediment recycling as a driver for postsubduction porphyry copper formation. *Sci. Adv.* **11**, eadx4474 (2025).

92. H. Li, Z. Yang, Y. Lu, Z. Hou, Redox state of subducted sediments controls porphyry copper mineralization along the Tethyan belt. *Nat. Commun.* **16**, 6456 (2025).

93. D. Canil, S. A. Fellows, Sulphide–sulphate stability and melting in subducted sediment and its role in arc mantle redox and chalcophile cycling in space and time. *Earth Planet. Sci. Lett.* **470**, 73–86 (2017).

94. Y.-C. Zheng, S.-A. Liu, C.-D. Wu, W. L. Griffin, Z.-Q. Li, B. Xu, Z.-M. Yang, Z.-Q. Hou, S. Y. O'Reilly, Cu isotopes reveal initial Cu enrichment in sources of giant porphyry deposits in a collisional setting. *Geology* **47**, 135–138 (2019).

95. J. Chang, A. Audétat, T. Pettke, The gold content of mafic to felsic potassic magmas. *Nat. Commun.* **15**, 6988 (2024).

96. X. Liu, L. Li, T. Xu, X. Xiong, J. Wang, Z. Wang, H. S. C. O'Neill, Gold solubility enhanced by H_2O in sulfur-bearing magma: Implications for gold partitioning and mineralization. *Geochim. Cosmochim. Acta* **393**, 170–181 (2025).

97. M. Gao, H. Xu, J. Zhang, S. F. Foley, Experimental interaction of granitic melt and peridotite at 1.5 GPa: Implications for the origin of post-collisional K-rich magmatism in continental subduction zones. *Lithos* **350-351**, 105241 (2019).

98. P. Condamine, E. Médard, Experimental melting of phlogopite-bearing mantle at 1 GPa: Implications for potassic magmatism. *Earth Planet. Sci. Lett.* **397**, 80–92 (2014).

99. C. Shu, S. F. Foley, I. S. Ezad, N. R. Daczko, S. S. Shcheka, Experimental melting of phlogopite websterite in the upper mantle between 1.5 and 4.5 GPa. *J. Petrol.* **65**, egae030 (2024).

100. A. Gale, C. A. Dalton, C. H. Langmuir, Y. Su, J.-G. Schilling, The mean composition of ocean ridge basalts. *Geochem. Geophys. Geosyst.* **14**, 489–518 (2013).

101. D. M. Ruscitto, P. J. Wallace, L. B. Cooper, T. Plank, Global variations in H_2O/Ce: 2. Relationships to arc magma geochemistry and volatile fluxes. *Geochem. Geophys. Geosyst.* **13**, Q03025 (2012).

102. U. Mann, M. W. Schmidt, Melting of pelitic sediments at subarc depths: 1. Flux vs. fluid-absent melting and a parameterization of melt productivity. *Chem. Geol.* **404**, 150–167 (2015).

103. Y. Wang, D. Prelević, S. Buhre, S. F. Foley, Constraints on the sources of post-collisional K-rich magmatism: The roles of continental clastic sediments and terrigenous blueschists. *Chem. Geol.* **455**, 192–207 (2017).

104. D. McKenzie, R. K. O'Nions, Partial melt distributions from inversion of rare earth element concentrations. *J. Petrol.* **32**, 1021–1091 (1991).

105. S. Erdmann, M. Pichavant, F. Gaillard, Mineral-melt vanadium oxybarometry for primitive arc magmas: Effect of hydrous melt composition on fO_2 estimates. *Contrib. Mineral. Petrol.* **179**, 39 (2024).

106. T. Plank, K. A. Kelley, M. M. Zimmer, E. H. Hauri, P. J. Wallace, Why do mafic arc magmas contain ~4 wt% water on average? *Earth Planet. Sci. Lett.* **364**, 168–179 (2013).

107. B. O. Mysen, D. Virgo, F. A. Seifert, The structure of silicate melts: Implications for chemical and physical properties of natural magma. *Rev. Geophys.* **20**, 353–383 (1982).

108. K. D. Putirka, M. Perfit, F. J. Ryerson, M. G. Jackson, Ambient and excess mantle temperatures, olivine thermometry, and active vs. passive upwelling. *Chem. Geol.* **241**, 177–206 (2007).

109. Z. Sun, X. Xiong, J. Wang, X. Liu, L. Li, M. Ruan, L. Zhang, E. Takahashi, Sulfur abundance and heterogeneity in the MORB mantle estimated by copper partitioning and sulfur solubility modelling. *Earth Planet. Sci. Lett.* **538**, 116169 (2020).

110. Z.-X. Anser Li, C.-T. A. Lee, The constancy of upper mantle fO_2 through time inferred from V/Sc ratios in basalts. *Earth Planet. Sci. Lett.* **228**, 483–493 (2004).

111. D. A. Singer, V. I. Berger, B. C. Moring, "Porphyry copper deposits of the world: Database and grade and tonnage models, 2008" (Open-File Report, 2008-1155, US Geological Survey, 2008).

112. W. Frisch, "Morphology across convergent plate boundaries," in *Encyclopedia of Marine Geosciences*, J. Harff, M. Meschede, S. Petersen, J. Thiede, Eds. (Springer Netherlands, 2013), pp. 1–7.

113. S. H. Kirby, S. Stein, E. A. Okal, D. C. Rubie, Metastable mantle phase transformations and deep earthquakes in subducting oceanic lithosphere. *Rev. Geophys.* **34**, 261–306 (1996).

114. H. S. C. O'Neill, The smoothness and shapes of chondrite-normalized rare earth element patterns in basalts. *J. Petrol.* **57**, 1463–1508 (2016).

115. S. Lallemand, D. Arcay, Subduction initiation from the earliest stages to self-sustained subduction: Insights from the analysis of 70 Cenozoic sites. *Earth Sci. Rev.* **221**, 103779 (2021).

116. M. Willbold, A. Stracke, Formation of enriched mantle components by recycling of upper and lower continental crust. *Chem. Geol.* **276**, 188–197 (2010).

117. D. Ben Othman, W. M. White, J. Patchett, The geochemistry of marine sediments, island arc magma genesis, and crust-mantle recycling. *Earth Planet. Sci. Lett.* **94**, 1–21 (1989).

118. W. M. White, B. Dupré, P. Vidal, Isotope and trace element geochemistry of sediments from the Barbados Ridge-Demerara Plain region, Atlantic Ocean. *Geochim. Cosmochim. Acta* **49**, 1875–1886 (1985).

119. H. R. Marschall, V. D. Wanless, N. Shimizu, P. A. E. Pogge von Strandmann, T. Elliott, B. D. Monteleone, The boron and lithium isotopic composition of mid-ocean ridge basalts and the mantle. *Geochim. Cosmochim. Acta* **207**, 102–138 (2017).
